# The cellular protein TIAR mediates rapid initiation of West Nile virus genome RNA synthesis

**DOI:** 10.1128/mbio.01947-26

**Published:** 2026-09-08

**Authors:** Jessica L. Siemer, Hsuan Liu, Jin Zhang, Dan Cui, Jeenah Jung, Philip J. Santangelo, Markus W. Germann, Margo A. Brinton

**Affiliations:** 1 Department of Chemistry, Georgia State University 1373, Atlanta, Georgia, USA; 2 Department of Biology, Georgia State University 1373, Atlanta, Georgia, USA; 3 The Wallace H. Coulter Department of Biomedical Engineering, Georgia Institute of Technology and Emory University 1371, Atlanta, Georgia, USA; Tsinghua University, Beijing, China

**Keywords:** TIAR, orthoflavivirus, West Nile virus, genome initiation

## Abstract

During the intracellular replication cycle of West Nile virus (WNV), genome RNA synthesis is initially inefficient but increases exponentially as viral replication complexes are sequestered in invaginations in the endoplasmic reticulum. In this study, we investigated the functional role of the cellular protein TIAR (T-cell intracellular antigen-related protein) in the transcription of WNV genome RNA. Close colocalization of cytoplasmic TIAR with viral double-stranded RNA was detected by a proximity ligation assay in WNV-infected cells. TIAR binds specifically to the WNV 3ʹ(−) SL but not to the complementary WNV 5ʹ(+) SL in *in vitro* RNA binding assays. Only the 3′ end of the WNV minus-strand RNA was enriched by immunoprecipitation of infected cell lysates with anti-TIAR antibody. Stable overexpression of TIAR in clonal A549 cells increased the ratio of intracellular viral plus-strand to minus-strand RNA in a dose-dependent manner. TIAR contains three RNA recognition motifs (RRMs). Biophysical data indicated that only RRM2 directly contacts RNA and that up to three TIAR molecules can bind cooperatively to the WNV 3ʹ(−) SL RNA. These data provide additional evidence that TIAR functions as a proviral host factor facilitating exponential amplification of WNV genome production in infected cells.

**IMPORTANCE:**

West Nile virus (WNV) is a mosquito-borne orthoflavivirus associated with increasing global human disease incidence. The molecular mechanisms underlying viral replication are not fully understood. In early stages of infection, viral genome transcription is inefficient; however, in late stages, viral genome transcription increases exponentially. T-cell intracellular antigen-related (TIAR) protein is a cellular protein that has been shown to interact with the 3′ end of the WNV negative-sense antigenomic RNA. We obtained data showing colocalization of cellular TIAR with viral replication complexes in infected cells and an increased ratio of intracellular genomic to antigenomic viral RNA in TIAR-overexpressing cells, and confirmed preferential binding of TIAR to the 3′ end of the WNV antigenome both *in vitro* and in infected cell extracts. We also demonstrated that multiple TIAR proteins can bind cooperatively to the WNV 3′(−) stem-loop RNA. These data provide supporting evidence for a model of TIAR-mediated rapid initiation of nascent genome RNA synthesis in infected cells.

## INTRODUCTION

West Nile virus (WNV) is a member of the genus *Orthoflavivirus* within the family *Flaviviridae* that spreads by a bird–mosquito–bird transmission cycle. Large mammals such as humans and horses are incidental dead-end hosts due to low viremia levels. The majority of human WNV infections are asymptomatic, but 20%–30% of those infected experience mild febrile illness and ~1% develop severe neurological disease that can be fatal (1). WNV is endemic in parts of Africa, the Middle East, southern Europe, south and central Asia, and Australia (2, 3) and since 1999 also endemic in the Americas (4). Recent WNV outbreaks in Europe and North America have been associated with an increased incidence of human neurological disease (5, 6). Currently, no WNV vaccines or specific therapeutic treatments beyond supportive therapy exist for humans (7).

The ~11 kb WNV genome is a single-stranded, positive-sense RNA with a 5ʹ type I cap and a highly structured 3ʹ untranslated region (UTR) without a 3ʹ poly(A) tail. After attachment to a cell, virions are endocytosed and uncoated, and the viral genome is translated. The genome then serves as a template for minus-strand RNA synthesis (8). Initially, low but similar amounts of viral plus-strand and minus-strand RNAs are produced (9). Exponential production of viral genomic plus-strand RNA begins after viral proteins remodel the endoplasmic reticulum (ER) membrane to create invaginations that contain viral replication complexes, viral double-stranded RNA (dsRNA), and putative host factors (10, 11). Some of the nascent genomes that exit through the pore of the virus replication complex vesicle (RCV) interact with ER membrane-associated viral structural proteins, resulting in the assembly and budding of nascent virions into the lumen of the ER (10, 12). Nascent immature viral particles are transported through the Golgi and processed by furin cleavage of the PrM protein into mature virions during transport through the cellular secretory pathway. Fusion of virion-containing vesicle membranes with the plasma membrane releases new virions from the infected cell (13).

Prior to initiation of minus-strand RNA synthesis, orthoflavivirus genomes cyclize via long-distance RNA–RNA interactions, and the 3′ and 5′ terminal nts of the genomes form conserved terminal stem-loop (SL) structures (14–16). The viral NS5 polymerase first binds to the 5′ SL of a cyclized genome and then moves to the nearby genome 3′ end to initiate transcription of a single antigenomic RNA. Nascent genome RNA is then efficiently transcribed from the antigenome in the context of this dsRNA by an uncharacterized mechanism. Viruses with small RNA genomes, such as orthoflaviviruses, encode a limited number of proteins and use cellular proteins to facilitate most phases of their intracellular replication cycles (17). We previously confirmed by RNase probing that the first 96 nts of the 3′ noncoding region of the WNV minus-strand RNA [WNV 3′(−) SL RNA] form a stem with three loop regions (17). We next detected four cell proteins with molecular masses of 108, 60, 50, and 42 kDa in S100 extracts from both uninfected and infected cells that bound to the WNV 3′(−) SL RNA in a UV-induced cross-linking assay (18). The 42 kDa protein was subsequently purified on an RNA affinity column and identified as T-cell intracellular antigen-related (TIAR) protein by peptide sequencing.

TIAR is a member of the RNA recognition motif (RRM) family of RNA-binding proteins with roles in regulating cellular alternative splicing and stress granule (SG) formation (19–22). The closely related cellular protein TIA-1 was also shown to bind the WNV 3′(−) SL RNA but with lower affinity than TIAR. A murine TIAR knockout stable cell line produced a lower yield of WNV than the control cells, and reconstitution of the knockout cells with TIAR increased the virus yield but to a lower level than that produced by the control cells. TIAR/TIA-1 are AU-rich element (ARE)-binding proteins (19), and the sequences of each of the three loops of the WNV 3′(−) SL RNA contain AREs. Mutant WNV genomes with the AU nts deleted or substituted with Cs in any of the three loops of the WNV 3′(−) SL RNA did not produce detectable virus after cell transfection. Deletion or mutation of a single nt at the edge of one of the loops had little effect on the efficiency of protein binding or virus production, but mutation of AU nts in the middle of a loop sequence reduced both *in vitro* TIAR/TIA-1-binding efficiency to WNV 3′(−) SL RNA and *in vivo* virus production. The ability of these mutant viruses to replicate at a low level suggested that the interaction between TIAR/TIA-1 and the viral 3′(−⁠) SL RNA is not required for initial low-level symmetric RNA replication but instead facilitates the subsequent asymmetric amplification of genome RNA from the minus-strand template.

In the present study, we further investigated the functional role of the cellular protein TIAR in WNV RNA replication as well as the characteristics of the viral 3′(−) SL RNA-TIAR interaction. Close colocalization of cytoplasmic TIAR with viral dsRNA was detected by a proximity ligation assay (PLA) in WNV-infected cells. TIAR showed specific binding to the WNV 3ʹ(−) SL but not to the complementary WNV 5ʹ(+) SL in *in vitro* RNA binding assays, and only the 3′ end of the WNV minus-strand RNA was enriched by immunoprecipitation of infected cell lysates with anti-TIAR antibody. Stable overexpression of TIAR increased the ratio of intracellular viral plus-strand to minus-strand RNA abundance in a dose-dependent manner. Biophysical characterization of the three TIAR RRMs indicated that only RRM2 directly contacts RNA and that multiple TIAR molecules can bind cooperatively to the WNV 3ʹ(−) SL RNA. The data support a mechanistic model of how TIAR facilitates exponential synthesis of WNV genomic RNA in infected cells.

## RESULTS

### TIAR colocalizes with viral dsRNA in WNV-infected cells

**Fig 1:**
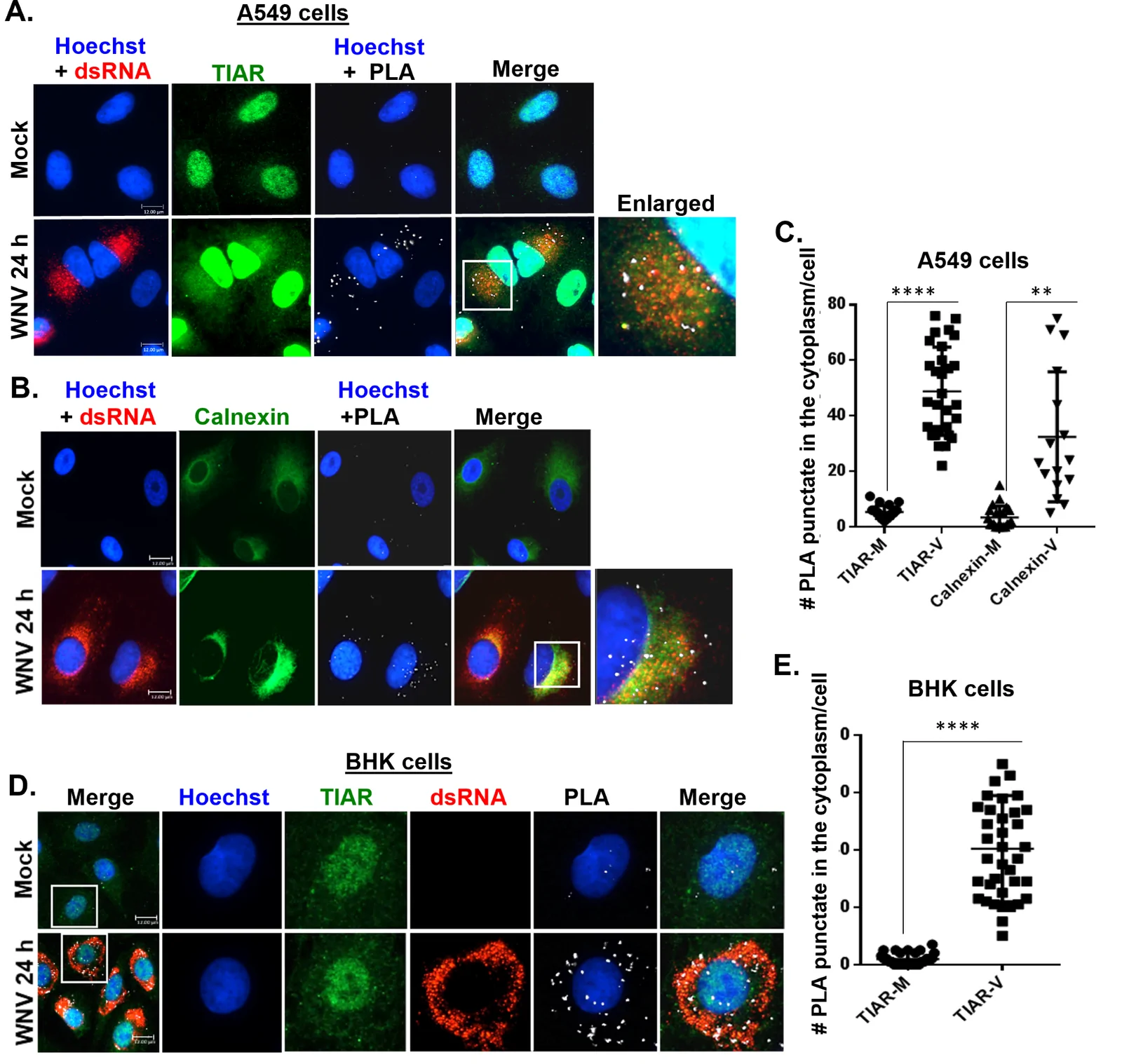
PLA detection of cytoplasmic TIAR and viral dsRNA colocalization in WNV-infected cells. (**A–C**) A549 or (**D** and** E**) BHK cells were mock-infected or WNV-infected (multiplicity of infection [MOI] of 5) and analyzed by PLA at 24 hpi. Cell nuclei were detected with Hoechst 33342 (blue). Cells were visualized with a 63× oil immersion objective on an Axio Observer Z1 widefield fluorescence microscope (Zeiss). Scale bars = 12 µm. (**A** and **D**) Anti-TIAR/anti-dsRNA and (**B**) anti-calnexin/anti-dsRNA PLA punctate (white) in mock-infected or WNV-infected cells. The number of cytoplasmic dsRNA PLA punctate in individual (**C**) A549 or (**E**) BHK cells was processed for quantification using Volocity 6.0.1 or ImageJ software. Scale bars, 9 µm in panel **A** and 20 µm in panels **B**, **C,** and **D** (µm/pixel [*X*] and µm/pixel [*Y*] were adjusted to 1). Statistical significance was determined by a Student’s *t*-test. **P < 0.01, ****P < 0.0001.

In uninfected cells, TIAR is primarily located in the nucleus, but some TIAR is diffusely distributed in the cytoplasm. If only a few molecules of TIAR are required within each viral RCV to enhance WNV genome synthesis, an indirect immunofluorescence assay (IFA) would not be able to detect colocalization of TIAR and dsRNA in the cytoplasm of infected cells. However, a PLA can detect close association (<40 nm apart) between two molecules inside cells even when these molecules are present at low levels (23). Cytoplasmic anti-TIAR/anti-dsRNA PLA punctae were detected in WNV-infected A549 cells at 24 h post-infection (hpi) (Fig. 1A). A PLA was also performed with anti-calnexin (an ER marker) and anti-dsRNA in infected cells and uninfected cells (Fig. 1B). Viral genome RNA is transcribed in ER membrane invaginations, and calnexin-dsRNA PLA puncta were previously detected in yellow fever virus-infected cells (24). Quantification of cytoplasmic PLA punctae per cell indicated that the average number of anti-TIAR/anti-dsRNA PLA punctae detected per infected A549 cell was greatly increased compared to the number (non-specific PLA) in mock-infected cells (Fig. 1C). The number of anti-calnexin/anti-dsRNA punctae per cell was lower than the number of anti-TIAR/anti-dsRNA punctae, consistent with a higher incidence of close TIAR−dsRNA colocalization than close calnexin−dsRNA colocalization. A high number of anti-TIAR/anti-dsRNA PLA punctae were also detected in the perinuclear region of WNV-infected BHK cells (Fig. 1D and 1E). The detection of cytoplasmic TIAR−dsRNA PLA punctae in both WNV-infected BHK and A549 cells indicated that TIAR and viral dsRNA are located close together in the perinuclear area in infected cells.

### Overexpression of TIAR enhances viral genome RNA production

**Fig 2:**
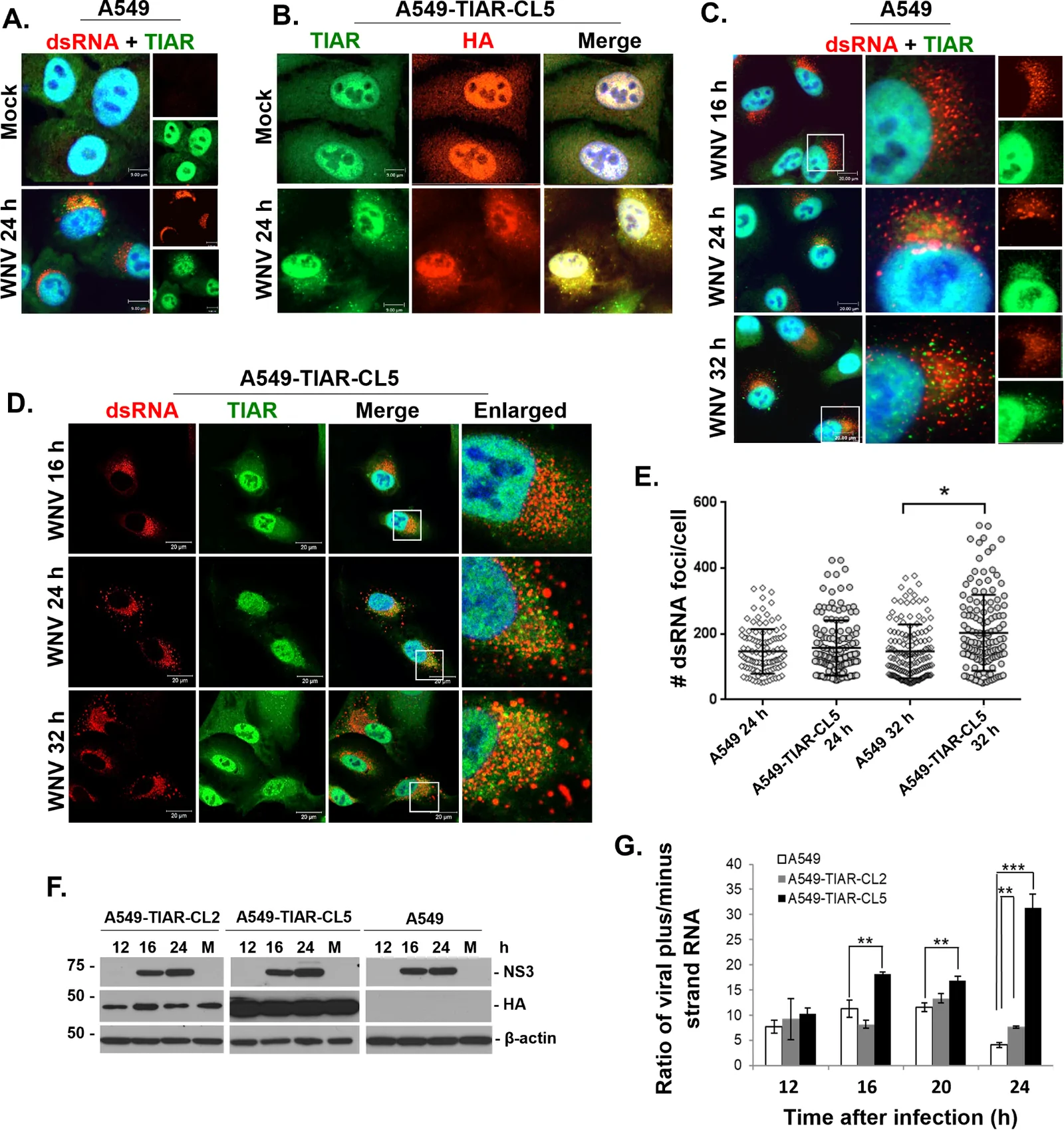
Effects of overexpression of TIAR on WNV replication. (**A**) A549 cells were mock-infected or WNV-infected (MOI of 5) and analyzed by IFA at 24 hpi using anti-TIAR and anti-dsRNA antibodies to detect the distribution of TIAR. (**B**) A549-TIAR-CL5 cells were mock-infected or WNV-infected (MOI of 5) and analyzed by IFA at 24 hpi using anti-TIAR and anti-HA antibodies to detect the distribution of total TIAR and of overexpressed HA-TIAR. (**C**) A549 or (**D**) A549-TIAR-CL5 cells were infected with WNV (MOI of 5) and analyzed by IFA at 16, 24, and 32 hpi using anti-TIAR and anti-dsRNA antibodies. (**E**) Quantification of dsRNA foci/cell in WNV-infected A549 and A549-TIAR-CL5 cells. (**F**) WT A549 cells and cells from two A549-TIAR clonal lines stably expressing HA-tagged TIAR were either mock-infected or infected with WNV (MOI of 5). Intracellular viral NS3 and HA-tagged TIAR were detected in cell lysates by western blotting with specific antibodies. β-Actin was used as a loading control. (**G**) The ratio of viral plus-strand RNA to minus-strand RNA was calculated from intracellular levels of viral RNA detected by qRT-PCR using strand-specific primers and probe. IFA images were acquired with a Zeiss Axio Observer Z1 widefield fluorescence microscope using a 63× oil immersion objective and processed for quantification using Volocity 6.0.1 or ImageJ software. Scale bars, 9 µm in panel **A** and 20 µm in panels **B**, **C**, and **D** (µm/pixel [*X*] and µm/pixel [*Y*] were adjusted to 1). Statistical significance was determined by a Student’s *t*-test (*P < 0.05, **P < 0.01, ***P < 0.001.

The effect of TIAR overexpression on intracellular viral RNA and protein levels and viral yields was analyzed. Clonal A549 cell lines stably expressing different levels of TIAR were established by transfecting cells with pEF6 plasmid DNA that expressed N-terminal HA-tagged TIAR under the control of an eEF-1α promoter and then cloning single antibiotic-resistant cells. TIAR is located mainly in the nucleus with some TIAR diffusely distributed in the cytoplasm in infected A549 cells with cytoplasmic TIAR colocalizing with viral dsRNA by 24 hpi (Fig. 2A). The intensity of the TIAR IFA signal was higher in uninfected and WNV-infected (MOI of 5) A549-TIAR-CL5 cells that express HA-TIAR, but the cellular distribution was like that observed in A549 cells (Fig. 2B). Stress granules were not detected in the cells overexpressing TIAR. A549 and A549-TIAR-CL5 cells infected with WNV (MOI of 5) were fixed and processed for IFA using anti-dsRNA and anti-TIAR antibodies. An increased number of dsRNA foci was detected in the infected A549-TIAR-CL5 cells compared to infected control cells (Fig. 2C through E). By 24 hpi, the size of some of the RCVs was larger in the infected A549-TIAR-CL5 cells compared to those in the control cells (Fig. 2C and D).

The effect of TIAR overexpression on viral protein and RNA levels was investigated in WNV (MOI of 5) infected A549, A549-TIAR-CL2, and A549-TIAR-CL5 cells. Similar levels of intracellular viral NS3 protein were detected in extracts from each of the cell lines at 16 h, and the level increased by 24 h, indicating that overexpression of TIAR does not have a negative effect on viral protein levels (Fig. 2F). Intracellular RNA was extracted from WNV-infected (MOI of 5) A549, A549-TIAR-CL2, and A549-TIAR-CL5 cells at 12, 16, 20, and 24 hpi, and viral plus-strand and minus-strand RNA levels, as well as control β-actin mRNA levels, were quantified by strand-specific qRT-PCR. At each time point analyzed, the Ct values for the control β-actin RNA from the three cell types were essentially identical, indicating that the total cell RNA amounts were consistently comparable. The plus-to-minus viral RNA ratios calculated for the three cell types were similar at 12 h but increased in the A549-TIAR-CL5 cells by 16 h and were significantly higher in these cells by 24 hpi (Fig. 2G). The results indicate that the presence of higher intracellular levels of TIAR preferentially enhanced viral genome RNA synthesis over minus-strand synthesis.

### TIAR preferentially interacts with the WNV 3ʹ(−) SL RNA

**Fig 3:**
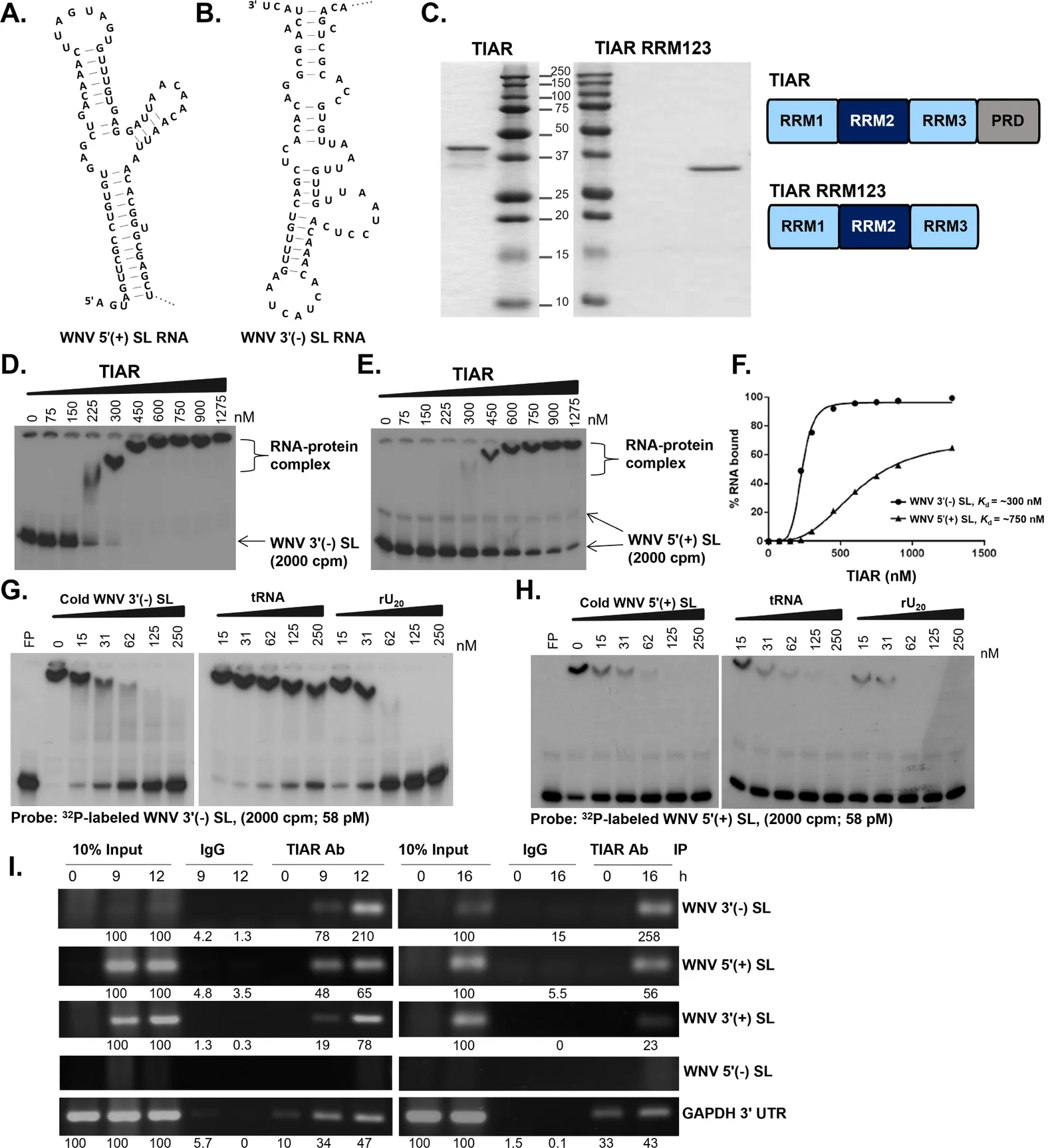
TIAR binds specifically to the WNV 3′(−) SL RNA *in vitro* and in WNV-infected cells. Predicted secondary structures of the (**A**) WNV 5′(+) SL and (**B**) WNV 3′(−) SL RNAs. (**C**) Purified TIAR and TIAR RRM123 proteins separated by SDS-PAGE and stained with Coomassie blue. Gel mobility shift assays using (**D**) ^32^P-WNV 3′(−) or (**E**) ^32^P-WNV 5′(+) SL RNA probes (2,000 cpm) and increasing amounts of purified TIAR (75−1,275 nM). (**F**) The quantified percentage of protein-bound RNA in the gels shown in (**D**) and (**E**) plotted against the TIAR concentration to generate Hill saturation binding curves. Competition gel mobility shift assays using a (**G**) ^32^P-WNV 3′(−) SL or (**H**) ^32^P-WNV 5′(+) SL RNA probe. Increasing concentrations of a specific competitor RNA, either a cold version of the probe or a 20-mer polyU (rU_20_), or a nonspecific competitor, tRNA, were added to the reactions containing ^32^P-labeled RNA (2,000 cpm; 58 pM) and 25 nM of TIAR. FP, free probe. (**I**) RNA−protein complexes were immunoprecipitated with anti-TIAR antibody from WNV-infected C3H/He MEF lysates prepared at 9, 12, and 16 hpi. Extracted RNA was amplified by RT-PCR to amplify the regions indicated. Each experiment was performed at least twice, and representative data are shown.

The sequences of the WNV 3′(−) SL and the 5ʹ(+) SL RNA are complementary, but the secondary structures of these two RNAs differ due to G-U base pairing (Fig. 3A and B). Gel mobility shift assays were conducted using ^32^P-labeled WNV 3ʹ(−) SL 74-mer RNA (Fig. 3A) or WNV 5ʹ(+) SL 75-mer RNA (Fig. 3B) and purified recombinant TIAR protein (Fig. 3C). Some TIAR binding was observed to the WNV 5ʹ(+) SL, which is expected due to the presence of a single-stranded ARE in the top loop; however, TIAR bound more efficiently to the WNV 3ʹ(−) SL probe (Fig. 3D) than to the WNV 5ʹ(+) SL RNA probe (Fig. 3E). Hill saturation binding curves were generated by plotting the percentage of bound RNA probe against the protein concentration (Fig. 3F). Next, TIAR binding specificity for the two RNA probes was analyzed by competition gel mobility shift assays. Although TIAR/TIA-1 is broadly classified as an ARE binding protein, previous studies identified U-rich sequences rather than AU-rich sequences as the preferred motifs of TIAR/TIA-1 (25–27). Therefore, we selected the rU_20_ sequence as a specific competitor. The two specific RNA competitors, cold WNV 3ʹ(−) SL and rU_20_, each efficiently competed with the ^32^P-labeled WNV 3ʹ(−) SL RNA probe, while the nonspecific competitor, tRNA, competed inefficiently (Fig. 3G). In contrast, cold WNV 5ʹ(+) SL, rU_20_ RNA, or tRNA efficiently competed with the ^32^P-labeled WNV 5′(+) SL RNA probe (Fig. 3H). The data indicated that TIAR binds specifically to WNV 3ʹ(−) SL RNA, but not to WNV 5ʹ(+) SL RNA in *in vitro* gel shift assays.

To determine whether TIAR interacts with the WNV 3ʹ(−) SL RNA in infected cells, TIAR−RNA complex immunoprecipitation assays were performed. Lysates from mock-infected or WNV-infected C3H/He MEFs were immunoprecipitated with anti-TIAR antibody or a control IgG. RNA present in the immunoprecipitants was extracted in the absence of an added RNase inhibitor and amplified by RT-PCR using primer sets specific for the terminal 5ʹ(+), 5ʹ(−), 3ʹ(+), or 3ʹ(−) regions of the viral RNAs or for the GAPDH mRNA 3ʹ UTR (Table S1). Consistent with data from a previous study (28), the amount of viral minus-strand RNA detected in the 10% input extracts was ~10-fold less than that of the viral plus-strand RNA. The intensity of each 10% input sample band was set at 100. At 9 hpi, the relative intensity of the WNV 3ʹ(−) RNA band was 78. The intensity of the WNV 3ʹ(−) RNA band increased to >200 at 12 and 16 hpi (Fig. 3I). In contrast, the relative band intensities for the WNV 5ʹ(+), 3ʹ(+), and 5′(−) RNAs were not enriched above those of their respective input bands at any of the times analyzed. The band intensity for the negative control GAPDH 3ʹ UTR RNA was also not enriched above that of the input band, and the IgG control antibody pulled down very low amounts of this RNA. These data indicate that TIAR interacts preferentially with the WNV 3ʹ(−) RNA in WNV-infected cells.

### Multiple TIAR proteins cooperatively bind to the WNV 3ʹ(−) SL through RRM2−RNA contacts

**Fig 4:**
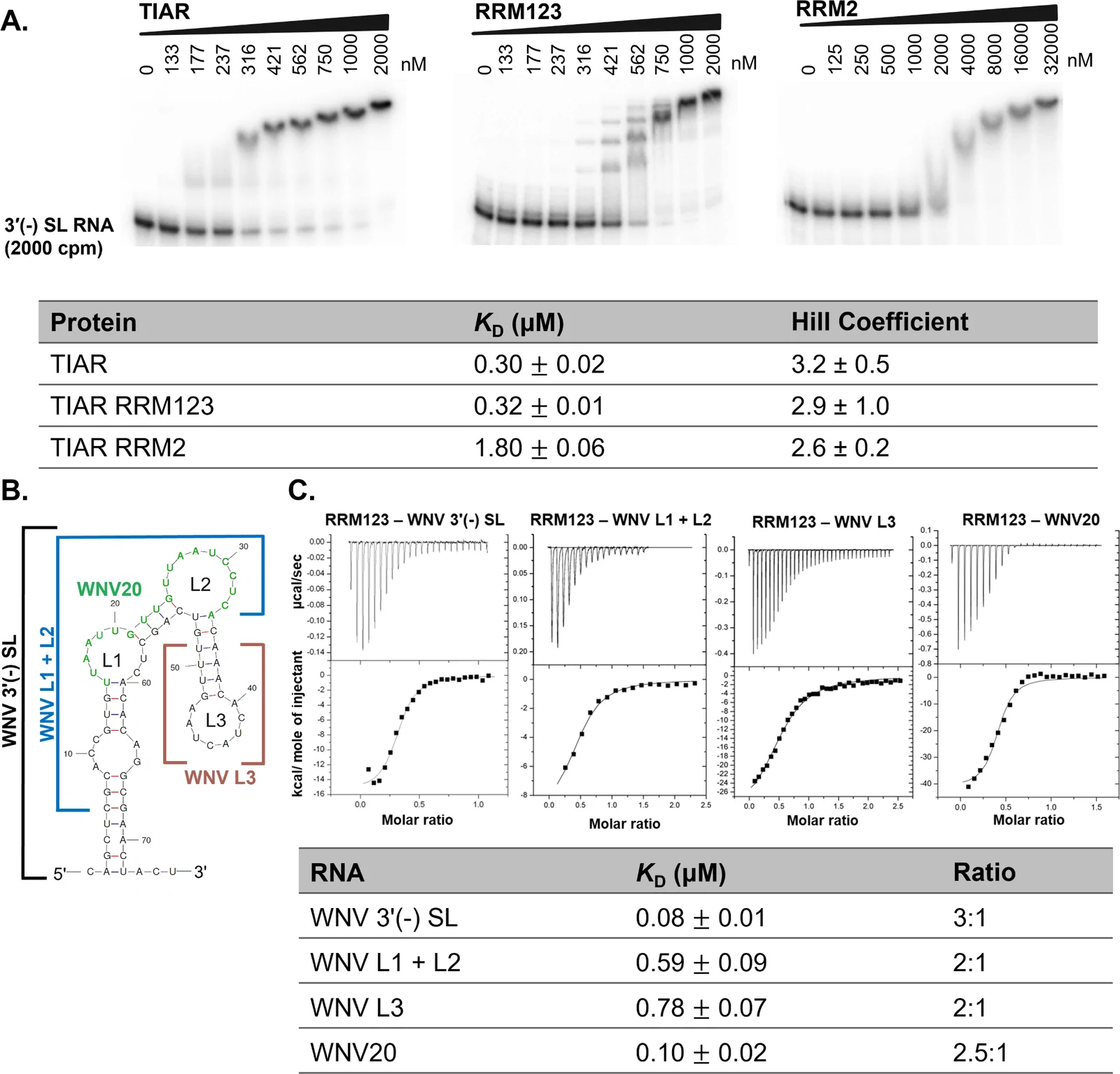
Multiple copies of TIAR RRM123 bind the WNV 3′(−) SL RNA loop regions *in vitro*. (**A**) Gel mobility shift assays using a ^32^P-WNV 3′(−) SL RNA probe (2,000 cpm) and increasing amounts of purified TIAR RRM2 (125−32,000 nM), TIAR RRM123 (133−2,000 nM), or TIAR (133−2,000 nM). Each experiment was repeated at least twice, and representative data are shown. The equilibrium dissociation constants (*K*_*D*_) and Hill coefficients were determined from gel shift assay data and fitted with the Hill equation using GraphPad Prism 6. (**B**) Secondary structure of the WNV 3′(−) SL RNA predicted using Mfold (33). RNA oligos (indicated by brackets) that contained the AU-rich nts of one or two of the loops of the WNV 3′(−) SL were commercially synthesized and used in biophysical assays. (**C**) Isotherms of RRM123 titrated with (left to right) WNV 3ʹ(−) SL, WNV 3′(−) L1 + L2 RNA, WNV 3′(−) L3 RNA, or WNV20 RNA. Dissociation constants and stoichiometric ratios derived from isothermal titration calorimetry (ITC) data were fitted with a one-site binding model.

We next investigated the binding mode of TIAR with the 3′(−) SL RNA. Previously, binding of the 3′(−) SL was mapped to TIAR RRM2 (18). Despite having similar protein structures, TIAR RRM1, RRM2, and RRM3 have been reported to differ in their RNA binding preferences, with TIAR RRM2 having the highest binding affinity for U-rich sequences (29, 30). Additional binding studies suggested that the related protein, TIA-1, forms protein−RNA contacts using multiple RRMs (31) and demonstrated that TIA-1 RRM3 can bind poly-U and poly-C sequences with micromolar affinity (32). To further characterize the regions of TIAR binding to the WNV 3ʹ(−) SL RNA, *in vitro* binding assays were performed using full-length TIAR, TIAR RRM123, and TIAR RRM2. Gel shift assay data obtained with a ^32^P-WNV 3′(−) SL RNA (Fig. 4A) were fitted to determine dissociation constants (*K*_*D*_) and Hill coefficients. The *K*_*D*_s of the full-length TIAR and RRM123 proteins were similar, while the ~6-fold higher *K*_*D*_ for RRM2 than for RRM123 indicated weaker binding by RRM2 alone. The Hill coefficients for all three proteins were ~3, and multiple protein−RNA complex bands of increasing size were detected for full-length TIAR, RRM123, and RRM2 as the RNA concentration increased. These data were indicative of multiple copies of each of these proteins binding cooperatively to an RNA. The data also showed that the C-terminal PRD domain of TIAR is not involved in RNA binding, but deletion of RRM1 and RRM3 decreased the affinity for the WNV 3′(−) SL RNA.

RRM domains have been reported to also participate in protein–protein interactions (34, 35). To determine whether the contributions of RRM1 and RRM3 to the binding affinity were due to direct RNA contacts or protein−protein interactions, we conducted binding experiments with RRM123, RRM1, or RRM3 and short RNA oligos using ITC (Fig. S1). ITC assays measure binding affinity, stoichiometry, and enthalpy of an interaction. RRM123 did not show detectable binding to 5-mer poly-A or 5-mer poly-C RNA oligos, indicating that neither RRM1, RRM2, nor RRM3 has high affinity for A-rich or C-rich sequences (Fig. S1A). Neither RRM1 nor RRM3 showed detectable binding to a 5-mer poly-U oligo, implying that neither of these RRMs binds with high affinity to U-rich sequences (Fig. S1B). These data suggested that RRM1 and/or RRM3 contribute to TIAR-binding affinity through protein–protein interactions rather than by directly binding RNA.

### TIAR proteins bind to each of the three loops in the WNV 3ʹ(−) SL RNA

We previously reported that mutation of mapped 3′(−) SL TIA-1/TIAR binding site nts that did not alter the nts required for NS5 binding in the complementary 5ʹ(+) SL RNA or the RNA secondary structures in a WNV infectious clone reduced the efficiency of genomic RNA production (36–38). Also, our previous gel shift data indicated that the 5 nt AU sequences of L1 and L2 of the WNV 3′(−) SL are required for efficient TIAR or TIA-1 binding, with mutation of the nts in L2 having the greatest effect on protein-binding affinity. Although mutation of the L3 nts did not decrease TIAR or TIA-1 binding in a gel shift assay, L1, L2, or L3 mutations in an infectious clone were lethal to the virus. To further characterize the interaction of TIAR with the WNV 3ʹ(−) SL RNA, ITC experiments were performed with RRM123 and four different 3ʹ(−) SL RNAs: the 75-mer WNV 3ʹ(−) SL, a 45-mer that forms L1 and L2, an 18-mer that forms L3, and a single-stranded 20-mer (WNV20) that contains the L1- and L2-binding sites (Fig. 4B and C). The stoichiometric ratios derived from the fitted binding curves indicated that three RRM123 proteins can bind to the WNV 3ʹ(−) SL RNA and two RRM123 proteins can bind to both the WNV 3ʹ(−) L1 +L2 RNA and the WNV 3ʹ(−) L3 RNA (Fig. 4C). However, the affinity of TIAR RRM123 was higher for the WNV 3ʹ(−) L1 +L2 RNA than for the WNV 3ʹ(−) L3 RNA, consistent with our gel shift data. ITC data obtained by titrating TIAR (Fig. S2A) or RRM123 (Fig. 4C) with WNV20 RNA yielded similar *K*_*D*_ values (Table 1) and thermodynamic parameters (Table S2), further confirming that the PRD domain does not contribute to RNA binding. WNV20 RNA titrations conducted with the individual RRMs (RRM1, RRM2, or RRM3) confirmed that only RRM2 bound with high affinity to the WNV20 sequence (Fig. S2B), with a *K*_*D*_ ~4× higher than the *K*_*D*_ for RRM123 (Table 1). Stoichiometric ratios determined from the fitted isotherms indicated that up to three RRM2s could bind to the WNV20 RNA (Table S2). The calculated stoichiometric ratios for TIAR or RRM123 binding to WNV20 RNA were 2.5, which is consistent with the larger size of these proteins limiting the number of proteins bound to an RNA that contained the L1 and L2 sequences (Table S2). These data further confirmed cooperative protein binding to a two binding site sequence, with only RRM2 directly contacting the RNA and the other RRMs enhancing binding through additional protein-protein interactions.

**TABLE 1:** Stoichiometry and affinity derived from titrations of RNA into protein as measured by ITC^*a*^

Protein	RNA	*K*_*D*_ (μM)	Ratio
TIAR	rU20	0.23 ± 0.02	2:1
	WNV20	0.12 ± 0.01	2.5:1
RRM123	rU5	11.10 ± 1.75	1:1
	rU15	0.03 ± 0.01	2:1
	rU20	0.191919 ± 0.01	2.5:1
	WNV20	0.10 ± 0.02	2.5:1
RRM2	rU5	8.70 ± 0.15	1:1
	rU15	0.34 ± 0.04	2:1
	rU20	0.25 ± 0.01	3:1
	WNV20	0.41 ± 0.05	3:1

^*a*^ Isotherms, fitted binding curves, and parameters are available in Fig. S2 and S3 and Table S2.

To map the length of the binding site on the RNA required for optimal protein binding, additional ITC experiments were conducted with poly-U sequences of varying lengths. Stoichiometric ratios (Table S2) derived from titration of rU_5,_ rU_15_, or rU_20_ oligos into RRM2 or RRM123 (Fig. S3) demonstrated that approximately the same number of RRM2 proteins or RRM123 proteins bound to sequences of the same length despite differences in protein size and provided further evidence that RRM2 predominantly contacts the RNA. Comparison of the stoichiometric ratios and *K*_*D*_ values for rU_15_ to those for rU_20_ suggested that ~7–8 nts are necessary for optimal binding of one RRM2 domain (Table 1).

As a second experimental method to investigate the binding mode of RRM123, steady-state fluorescence anisotropy measurements were performed by titrating a fluorescently tagged rU_20_ sequence (FrU_20_) into RRM123 or RRM2. The data indicated that the anisotropy at steady state of the RRM123-FrU_20_ complex was markedly higher than the anisotropy of the RRM2-FrU_20_ complex (Fig. S4). Since anisotropy is dependent on the complex size and each RRM domain has a similar molecular mass, it was expected that three copies of RRM2 bound to an RNA sequence would produce anisotropy measurements that were similar to those for one copy of RRM123 bound to the same RNA sequence, with each of the three RRM domains contacting the RNA. However, this was not the case, and the data further supported the ability of multiple RRM123 proteins to bind to rU_20_, with each protein using only RRM2 to contact the RNA.

### Electrostatic interactions facilitate cooperative RNA binding of RRM123 or RRM2 to RNA

Electrostatic and hydrophobic maps for TIAR RRM1, RRM2, and RRM3 were generated in UCSF Chimera (39) and used to visualize potential interaction surfaces (Fig. S5). Based on the crystal structure of RNA-bound TIA-1 RRM2 (PDB: 5ITH), the RNA is predicted to bind to the β-sheet surface of TIAR RRM2 (40). The TIAR β-sheet surface is positively charged, while the corresponding surfaces on RRM1 and RRM3 are neutral or even repulsive to nucleic acids. The α-helical regions of RRM2 are predominantly negatively charged, which under low-salt conditions would be expected to reduce the binding affinity of additional RRM2s that are in proximity. Each of the three RRMs contains hydrophobic patches that could support protein–protein contacts between multiple RNA-bound copies of RRM123.

**Fig 5:**
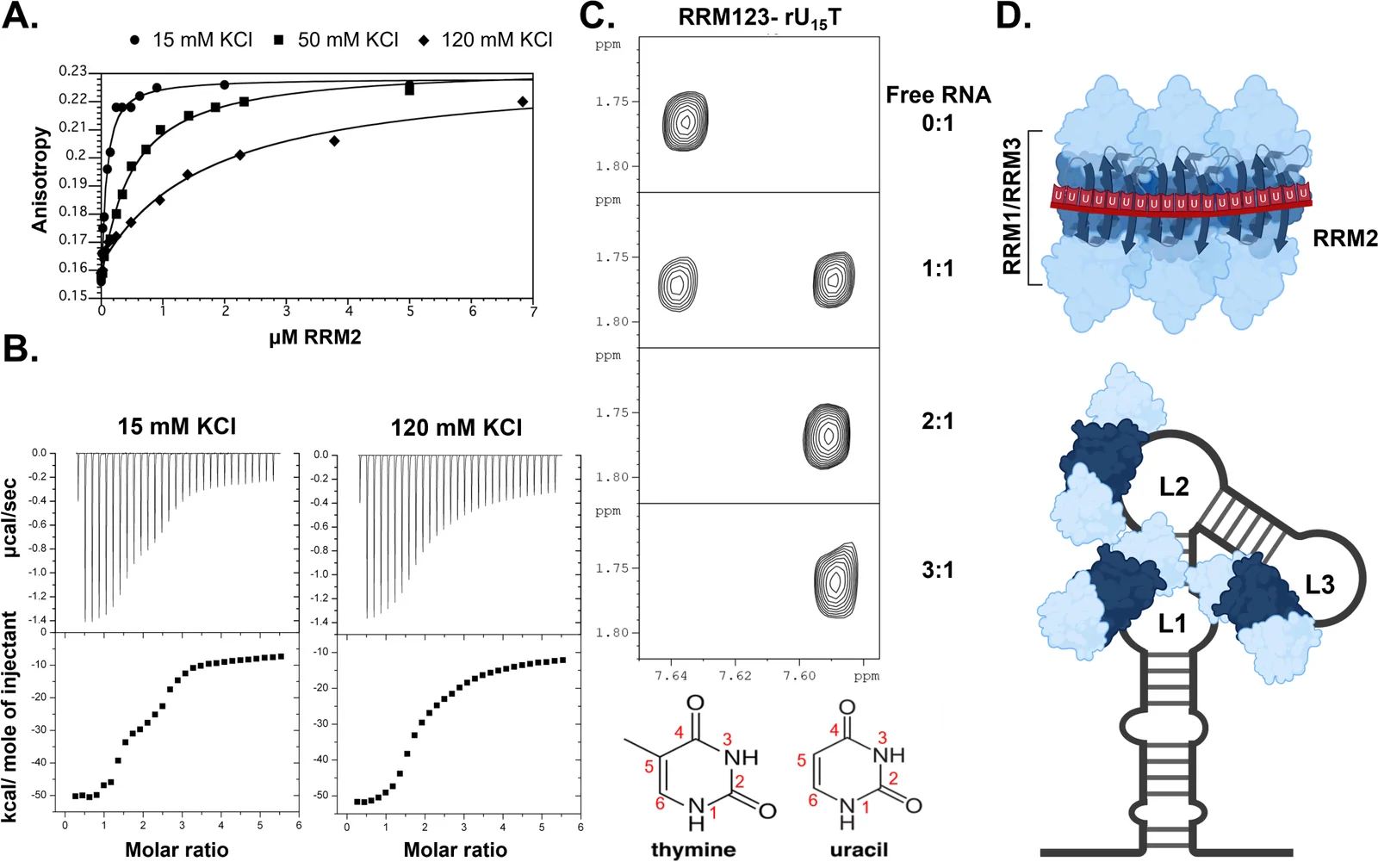
The binding of RRM123 to RNA is salt-dependent and cooperative. (**A**) Anisotropy measurements of the RRM2-FrU_6_ complex at low (15 mM KCl), medium (50 mM KCl), and high (120 mM KCl) salt conditions. (**B**) ITC experiments were performed under low- (left isotherm) and high-salt (right isotherm) buffer conditions. RRM123 (75 µM) was titrated into rU_20_ (5 µM). (**C**) TOCSY of rU_15_T (50 µM) titrated with increasing amounts of RRM123 in a 1:1, 2:1, and 3:1 ratio of protein to RNA. A chemical shift of the T_16_Met-H6 cross-peak was observed upon protein binding. (**D**) Binding modes of RRM123 with rU_20_ (top) or the WNV 3′(−) SL (bottom). ITC and anisotropy data support that only RRM2 directly contacts the RNA and ~7–8 nts provide an optimal binding footprint for each RRM2. Three copies of RRM123 can bind to the U-rich regions of L1, L2, and L3, with RRM2 directly contacting the RNA and RRM1 and RRM3 possibly stabilizing the interaction through electrostatic interactions. RRM2 is depicted in dark blue, and RRM1/RRM3 is depicted in light blue.

To assess the effects of salt concentration on RRM2 binding to RNA, fluorescence anisotropy experiments were conducted with a short fluorescently labeled oligo, FrU_6_, that ensured that a 1:1 protein–RNA complex formed with no contributions from protein–protein contacts. The slightly longer rU_6_ RNA than the rU_5_ used in the experiments described above was chosen to mitigate potential effects of the fluorophore. Binding curves were recorded under low (15 mM), medium (50 mM), and high (120 mM) salt conditions (Fig. 5A), and *K*_*D*_ values derived from curve fitting for the three conditions were approximately 0.06 μM, 0.54 μM, and 1.30 μM, respectively. The anisotropy-derived binding constant for RRM2-FrU_6_ at 120 mM KCl (*K*_*D*_ = 1.30 µM) differed from the ITC-determined binding constant for RRM2-rU_5_ at 100 mM NaCl (*K*_*D*_ = 8.70 µM), mainly due to the additional U residue and its charge. A competition assay measured by fluorescence anisotropy further supported this conclusion by showing that rU_5_ poorly competed with FrU_6_ (Table S3). We then investigated the effects of salt in the context of multiple proteins by conducting reverse ITC experiments (41) in which RRM123 was titrated into the sample cell containing rU_20_ in low- or high-salt conditions (Fig. 5B). In low salt, two distinct binding events with different affinities were detected with the formation of a 2:1 RRM123-rU_20_ complex followed by formation of a 3:1 complex with the third RRM123 binding having a decreased affinity. Under these conditions, the first 2 RRM123 molecules bind with high affinity to rU_20_, while the third RRM123 molecule is left with only a short U tract for binding, resulting in the distinct binding curve observed. In high salt, the different binding events were not resolved due to a combination of decreased affinity to the RNA under high-salt conditions, as previously shown in the RRM2 anisotropy experiments, and the reduction of electrostatic repulsion between the proteins bound on the RNA.

Next, we used 2D NMR to further investigate whether RRM binding is cooperative using a rU_15_ RNA with a 3′ thymidine (rU_15_T). The unique signal from the T methyl group was used to distinguish the 5′ end from the 3′ end due to the TOCSY cross-peak observed between the T H6 and methyl protons (Fig. 5C). The addition of RRM123 to the RNA shifted approximately half of the signal upfield, yielding two distinct cross-peaks when a 1:1 ratio was used and shifted all the signal upfield when either a 2:1 or a 3:1 ratio was used. Since binding was monitored through the RNA peaks, it was not possible to detect excess protein. Based on the previous ITC data, at the 3:1 protein–RNA titration ratio, two RRM123s are expected to be bound to rU_15_T with excess free RRM123 present. At a 1:1 protein–RNA ratio, only two major species are predicted to exist: unbound rU_15_T and the RNA bound to two proteins. At the 1:1 ratio, the chemical shifts of the two cross-peaks corresponded with those expected for the free RNA and RNA in complex with two RRM2 proteins.

The ITC data indicated that all three loops of the WNV 3′(−) SL RNA can bind TIAR, but that RRM123 has a higher affinity for L1 + L2 than for L3. Two RRM123 proteins can bind to an RNA containing the L1 and L2 sequences. The L3 hairpin alone can accommodate two RRM123 proteins, possibly due to additional binding site accessibility from unpairing of the stem. Mutation of the L3 nts would not abolish TIAR binding to the WNV 3′(−) SL RNA but would reduce the number of proteins bound from three to two, thus decreasing protein–protein stabilization. The ITC, anisotropy, and NMR data support the hypothesis that three TIAR proteins can cooperatively bind to the WNV 3′(−) SL RNA, with one RRM123 binding to each of the loops (Fig. 5D).

## DISCUSSION

Our previous and current biological and biophysical data provide strong support for the involvement of TIAR, and likely also TIA-1, in facilitating the rapid synthesis of genomic RNA from minus-strand templates inside viral RCVs. The biological data indicate that the interaction between TIAR and the WNV 3′(−) SL RNA is specific, that TIAR colocalizes with dsRNA in RCVs, and that TIAR overexpression increases the number of dsRNA foci and the ratio of genomic to antigenomic RNA in a dose-dependent manner in infected cells. The biophysical data provide insight into the nature and function of this viral RNA–cellular protein interaction and support a model in which three TIAR proteins cooperatively bind to the WNV 3′(−) SL RNA, with one TIAR binding to each of the three loops. Another RRM-containing protein, heterogeneous nuclear ribonucleoprotein C1/C2 (hnRNP C), was previously shown to form protein multimers and cooperatively bind tandem RNA sites with different affinities (42). Additionally, tandem sites within the p53 mRNA 3′ UTR were shown to bind TIA-1 and enhance phase separation (43). It has been hypothesized that cooperative binding of proteins to tandem sites in an RNA can enhance the specificity and affinity of an RNA-binding protein (44).

**Fig 6:**
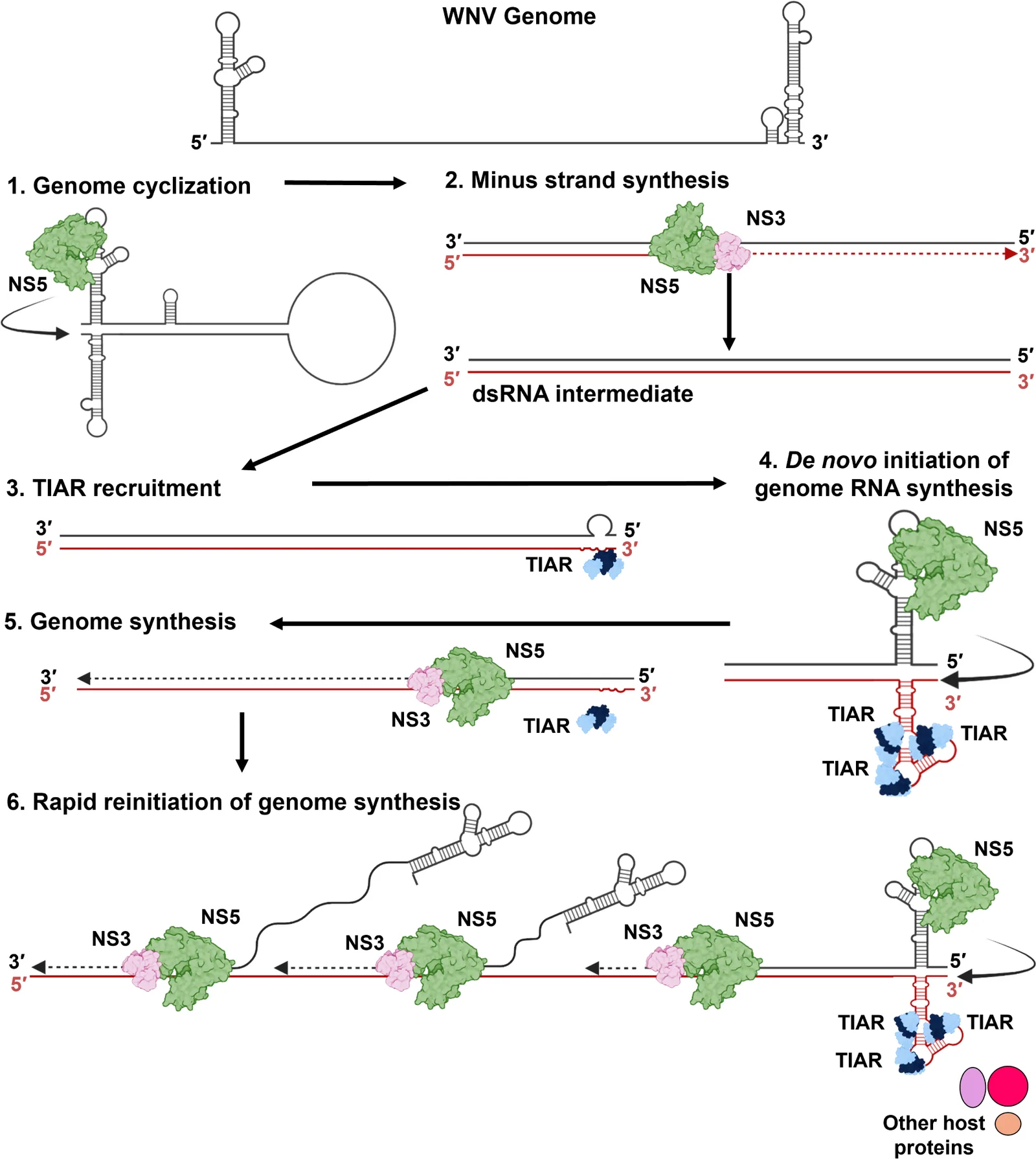
Proposed mechanism for rapid reinitiation of genome synthesis. 1. Cyclization of the genomic RNA (black) through long-range complementary RNA interactions initiates the process of orthoflavivirus genome replication. The viral polymerase, NS5 (green), is recruited to the 5′(+) SL and initiates synthesis of the negative-sense template strand (red) at the 3′(+) terminal -CU. 2. A dsRNA intermediate is formed from the positive-sense genome and antigenome, the negative-sense template strand. 3. TIAR binds to AU-rich regions at the 3′(−) end, facilitating the formation of both the 5′(+) and 3′(−) SLs. 4. NS5 is recruited to the 5′(+) SL and *de novo* initiates genome replication on the 3(−) terminal -CU. 5. The viral helicase, NS3 (pink), unwinds double-stranded regions of SL structures during genome replication, disassociating the TIAR proteins from the template strand. 6. At late-stage infection in the RCVs, rapid reinitiation of genome synthesis occurs by TIAR and potentially other host proteins, rapidly facilitating SL formation at the 5′(+) end of the nascent genomic RNA strand by stabilizing the complementary 3′(−) SL of the template strand, enabling continuous recruitment of NS5 to the 5′(+) SL.

Although all the steps and components involved in the molecular mechanism facilitating the rapid reinitiation of nascent orthoflavivirus genome RNA synthesis from a minus-strand template inside RCVs in infected cells are currently unknown, important steps likely include the initial release of the 3ʹ end of the minus-strand template RNA from the most recent genome RNA product, refolding of the template 3ʹ(−) SL, folding of the 5′(+) SL of the most recent genome product, binding of NS5 to the most recent genome product 5′(+) SL, transfer of NS5 to the 3′ end of the minus strand followed by *de novo* initiation on the 3′(−) CU (Fig. 6) (45–49). A recent study showed that the 5′ and 3′ SLs of a DENV genome readily form a duplex that mimics the replication intermediate under *in vitro* conditions. Although both NS5 and NS3 bound to this dsRNA intermediate, NS3 was unable to unwind it. Also, an annealed duplex formed by a 135 nt RNA from the 5′ end of the DENV genome and its complementary negative strand could not be unwound by NS3 under the *in vitro* conditions tested (50). The authors concluded that NS3 requires a 3ʹ overhang for efficient helicase activity and that additional cofactors are needed for separation of the minus strand from the plus strand and the subsequent formation of the terminal SLs. We hypothesize that AU breathing in tandem regions at the 3′(−) end of the minus-strand RNA in the context of the double-stranded RNA formed by the transcription of the most recent genome (+) RNA would allow cooperative recruitment of three TIAR proteins, facilitating the subsequent formation of the 3′(−) SL and the complementary 5(+) SL. The NS5 polymerase would bind to the re-formed 5′(+) SL and then move to the 3′(−) CU and *de novo* initiate transcription of a genome RNA. The unwinding activity of the viral helicase, NS3, would dissociate the TIAR proteins from the 3′ end of the minus-strand template.

Repeated recruitment of three TIARs to the AREs near the 3′ end of the minus-strand template when it is paired with the 5′ end of the most recently transcribed nascent genome would facilitate rapid refolding of the 3′(−) SL, allowing the 5′ SL of the most recent nascent genomic strand to fold and initiation of a new genome RNA to occur. Base pair breathing in AU-rich regions was previously suggested as a possible mechanism for homologous recombination during brome mosaic virus positive-strand synthesis (51). This plant RNA virus does not encode a helicase and may recruit the cellular Ded1p DEAD-box RNA helicase to bind the 3′-end of the viral minus-strand RNA and unwind local structures prior to initiation of the transcription of a nascent plus-strand RNA (52). In addition to the 42 kDa TIAR protein, three yet unidentified cellular proteins (108, 60, and 50 kDa) were immunoprecipitated from cellular S100 extracts by the WNV 3′(−) SL RNA (18) that may also be involved in facilitating genome transcription from the minus-strand template. While the activity of the WNV helicase, NS3, may be sufficient to unwind the dsRNA and dissociate the TIAR proteins from the 3′(−) SL, it is possible that one of the additional cellular proteins could be a cellular helicase.

## MATERIALS AND METHODS

### Cell lines

All cells were maintained at 37°C in a 5% CO_2_ atmosphere. Baby hamster kidney, strain WI2 (BHK) (obtained from Dr. Tadeusz Wiktor, Wistar Institute, Philadelphia) cells, and C3H/He MEFs were maintained in minimal essential medium (MEM; Life Technologies) supplemented with 5% fetal bovine serum (FBS) and 10 μg/mL of gentamicin. Human lung carcinoma A549 cells (purchased from ATCC) were maintained in F-12K Nutrient Mixture (Kaighn’s) (Life Technologies) supplemented with 10% FBS and 100 units/mL of penicillin and 100 μg/mL of streptomycin.

Clonal A549 cell lines stably expressing TIAR were generated by transfecting cells with pEF6-HA-TIAR (eEF-1a promoter) DNA using Lipofectamine 2000 (Invitrogen) and then incubating cells with blasticidin (10 μg/mL). Single-cell clones were selected, expanded, and maintained in drug-containing media. Two of the clones, A549-TIAR-CL 2 and 5, were selected for use in experiments.

### Viruses

A stock of WNV, strain Eg101 (obtained from Dr. Hilary Koprowski, Wistar Institute, Philadelphia), was prepared by infecting a confluent monolayer of BHK cells at an MOI of 0.1 and harvesting culture fluid at 32 hpi. Clarified culture fluid (1 × 10^8^ PFU/mL) was aliquoted and stored at −80°C.

### Plaque assay

Viral infectivity titers were determined by infecting monolayers of BHK cells in 6-well plates in duplicate with serial 10-fold dilutions of a virus sample. After adsorption for 30 min at 37°C, the virus inoculum was removed, and the cells were overlaid with MEM containing 2.5% FBS and 0.5% SeaKem ME agarose (Bio-Whittaker Molecular Applications) and incubated for 72 h at 37°C in a 5% CO_2_ atmosphere. After removing the agarose plugs, cells were stained with 0.05% crystal violet in 10% ethanol.

### Indirect immunofluorescence assay

Cells grown to ~80% confluency on 12 mm diameter coverslips (Fisher Scientific) in 24-well plates were infected with WNV at an MOI of 5. At 24 h after WNV infection or mock infection, the cells were fixed with 4% paraformaldehyde in phosphate-buffered saline (PBS) for 10 min at room temperature (RT), permeabilized with 0.1% Triton X-100 in PBS, washed with PBS, and blocked with 5% horse serum in PBS for 1 h at RT. Cells were then incubated with primary antibodies in PBS plus 5% horse serum for 1 h at room temperature, washed, incubated with appropriate secondary antibodies (Alexa Fluor 488 or 594 conjugated donkey anti-mouse, anti-rabbit, or anti-goat IgG antibody; Invitrogen) and 0.5 μg/mL of Hoechst 33258 dye (Invitrogen) in PBS plus 5% horse serum for 1 h at RT and then washed. Primary antibodies used were mouse anti-dsRNA (1:1,000; Jena Bioscience, Jena, Germany), goat anti-TIAR (C-18) (1:200; Santa Cruz Biotechnology), rabbit anti-calnexin (1:400; Cell Signaling), rabbit anti-GAPDH (1:2,000; Sigma), and mouse anti-HA (1:400; BioLegend). Images were obtained with a Zeiss LSM 510 confocal laser scanning microscope or with 63× or 40× oil immersion objectives on a Zeiss Axio Observer Z1 widefield fluorescence microscope. For protein colocalization analyses, image stacks from the widefield microscope were recorded at 200 nm intervals and subjected to “iterative deconvolution” using Volocity software (PerkinElmer).

To quantify viral dsRNA foci in WNV-infected A549 and A549-TIAR-CL-5 cells, images were obtained on a Zeiss widefield fluorescence microscope and were deconvoluted using Volocity software (PerkinElmer). The nuclei were first identified using the Find Object command to detect the Hoechst 33258 signal above the background threshold. Then, the dsRNA foci signals were detected using the Find Object command with the threshold intensity determined from the mock-infected cells, the Separate Touching Objects command with the object size guide set to 0.1 µm^2^, and the Exclude Object by Size command set to objects smaller than 0.02 µm^2^. The Compartmentalize command was then used to count dsRNA foci associated with each nucleus.

### Proximity ligation assay

Cells grown to ~80% confluency on 10 mm diameter coverslips in 24-well plates were infected with WNV at an MOI of 5. At 24 hpi, cells were fixed with 1% paraformaldehyde in PBS for 10 min at RT, permeabilized with 0.1% Triton X-100 in PBS, washed with PBS, and blocked with 5% horse serum in PBS for 30 min at 37°C. The cells were first incubated with two primary antibodies in PBS containing 5% horse serum for 30 min at 37°C and then washed with Duolink wash solution (Olink Bioscience). The same primary antibodies as described above for IFA were used at twofold lower concentrations. The cells were then incubated with complementary PLUS and MINUS oligonucleotide-labeled PLA probes diluted in Duolink *In Situ* Antibody Diluent (Olink Bioscience) for 1 h at 37°C and washed with Duolink wash solution. Ligation and rolling-circle amplification reactions were performed according to the manufacturer’s protocol. The cells were incubated in PBS with appropriate secondary Alexa Fluor antibodies for 30 min at 37°C and then with Hoechst dye for 5 min at 37°C. Images were acquired with a Zeiss widefield fluorescence microscope. The number of cytoplasmic PLA punctae per cell was quantified using Volocity software (PerkinElmer) for 20–40 representative cells (53). The PLA punctae were identified by the “find objects using standard deviation (SD) intensity,” then separated into individual punctae using the “separate touching objects” tool and filtered based on size. Punctae located in or over the nucleus were excluded from the calculation. GraphPad Prism 6 was used to plot the data.

### Cloning, expression, and purification from *E. coli*

Human TIAR, individual TIAR RRM cDNAs, and TIAR RRM123 cDNA were amplified from plasmid pMT2-TIAR (a gift from Paul Anderson, Harvard Medical School) and subcloned into pGEX-6P-1 (GE Healthcare), which contains a T7 promoter and cDNA for a fused N-terminal glutathione S-transferase (GST)-tag. All RRM subunit constructs include the RNA-contact residues in their C-terminal flexible linker regions: RRM123 (residues 1–294), RRM1 (residues 1–91), RRM2 (residues 93–196), and RRM3 (residues 196–294) (29). All constructs were transformed and expressed in Rosetta 2(DE3) pLysS cells (Novagen).

For full-length TIAR, transformed cells were grown in LB medium containing carbenicillin (50 μg/mL) and chloramphenicol (34 μg/mL) to an optical density at 600 of 0.8 at 37°C. The cells were induced overnight at 20°C with isopropyl-D-thiogalactoside (0.5 mM) and harvested. Individual GST-tagged RRMs and RRM123 were separately expressed as previously reported (18). Cell pellets were resuspended in lysis buffer (Cellytic Express [Sigma], 1 mM DTT, and complete EDTA-free protease inhibitor cocktail [Roche] in PBS) and incubated at 37°C until lysed and clarified by centrifugation at 10,000 × *g* for 20 min. For the purification of individual GST-tagged RRMs and RRM123, clarified lysates were incubated with 0.2% polyethylenimine at 4°C for 30 min, followed by ammonium sulfate precipitation at 75% saturation at 4°C for 30 min. The resulting pellets were resuspended in PBS. Clarified cell lysates containing full-length GST-TIAR or resuspended pellets containing GST-RRM or RRM123 were incubated at 4°C overnight with glutathione Sepharose 4B matrix in the Bulk GST Purification Module (GE Healthcare) according to the manufacturer’s protocol. The protein-bound Sepharose was washed and then incubated with PreScission Protease (GE Healthcare) in cleavage buffer (50 mM Tris-HCl [pH 7.0], 150 mM NaCl, 1 mM EDTA, and 1 mM DTT) at 4°C. The cleaved protein was eluted with cleavage buffer. The collected fractions for the individual RRM subunits were concentrated and further purified using a Superdex-75 preparative column. RRM123 was separated using a centrifugal concentrator (MWCO 50 kD, Amicon). The purified proteins were separated by 13.5% SDS-PAGE (37.5:1 acrylamide/bis-acrylamide) and stained with Coomassie blue. Protein folding was validated using ^1^H NMR (Fig. S6).

### *In vitro* transcription of ^32^P-labeled RNA probes and unlabeled RNA transcript**s**

The DNA templates used to transcribe the WNV 3ʹ(−) SL RNA (75 nts) and WNV 5ʹ(+) SL RNA (74 nts) probes were PCR amplified from the previously constructed plasmid p5ʹNCR (36). The forward primer for WNV 3ʹ(−) SL RNA was NCR75R, and the reverse primer was NCR75F (Table S1). The forward primer for WNV 5ʹ(+) SL RNA was 5ʹ-[T7]AGTAGTTCGCCTGTGTGAGC-3ʹ, and the reverse primer was 5ʹ-AGCTCGCACTGTGTTAATTGTTG-3ʹ. The PCR products were gel-purified using a MiniElute PCR Purification Kit (QIAGEN) and dissolved in RNase-free water. ^32^P-labeled RNAs were *in vitro* transcribed using a MAXIscript T7 Transcription Kit (Ambion) following the manufacturer’s instructions with 50 μCi [α-^32^P] GTP (PerkinElmer), 30 μM GTP, and 0.5 mM CTP, UTP, and ATP. RNA transcripts were electrophoresed on a 6% denaturing polyacrylamide gel (19:1 acrylamide/bis-acrylamide with 6 M urea). The wet gel was autoradiographed, and the ^32^P-labeled RNA band was excised. RNA was eluted by rocking overnight at 4°C in elution buffer (0.5 M NH_4_OAC, 1 mM EDTA, and 0.2% SDS), filtered through a 0.45 μm cellulose acetate filter unit (Millipore), ethanol precipitated, and resuspended in water. Unlabeled WNV 3ʹ(−) SL RNA and WNV 5ʹ(+) SL RNA were *in vitro* transcribed using a high-yield MEGAshortscript Kit (Ambion) according to the manufacturer’s protocol and purified in water using NucAway Spin Columns (Ambion). The sizes were confirmed by electrophoresis on a 6% denaturing polyacrylamide gel with GelStar Nucleic Acid Gel Stain (Lonza).

### Gel mobility shift assay and competition gel mobility shift assay

^32^P-labeled RNA probe diluted in binding buffer (5 mM HEPES [pH 7.5], 25 mM KCl, 2 mM MgCl_2_, 0.1 mM EDTA, 2 mM DTT, and 3.8% glycerol) was denatured at 85°C for 10 min and slowly renatured (0.1°C/s) to 20°C. The renatured RNA probe (2,000 cpm; ~58 pM) and different concentrations of purified TIAR (75–1,275 nM) were incubated at RT for 30 min in a final volume of 10 μL of binding buffer containing RNase inhibitor and 1 mM DTT. The RNA–protein complexes were electrophoresed on a 9% nondenaturing polyacrylamide gel (29:1 acrylamide/bis-acrylamide) at 4°C. Gels were dried and visualized by autoradiography or with a phosphorimager (Fuji). The binding activities of the WNV 3′(−) SL RNA and WNV 5′(+) SL RNA were assessed in gel mobility shift assays using the same dilutions of a single batch of purified TIAR. For competition gel mobility shift assays, increasing concentrations of specific or non-specific competitor RNAs were added to reactions containing constant concentrations of purified TIAR and ^32^P-labeled RNA probe. The RNA–protein complexes generated were analyzed as described above for the gel mobility shift assay. The intensities of the free RNA probe bands in gel mobility shift assays were quantified using Multi Gauge V3.1 software. The percent RNA bound was calculated by determining the percent decrease in the amount of free RNA probe with increasing protein concentration, and these values were plotted against protein concentration to generate an average theoretical Hill saturation binding curve using GraphPad Prism 6 (54–57).

### Immunoprecipitation of intracellular RNA–protein complexes

RNA–TIAR complexes were immunoprecipitated from infected cell lysates using an anti-TIAR antibody and a Magna RNA-binding protein immunoprecipitation (RIP) kit (Millipore). Confluent monolayers of C3H/He MEFs in T-150 flasks were mock-infected or infected with WNV at an MOI of 5. At different times after infection, cells were washed twice with ice-cold PBS, scraped into 5 mL of ice-cold PBS, centrifuged at 1,800 × *g* for 5 min at 4°C, resuspended in 100 μL of complete RIP Lysis Buffer (Millipore), incubated on ice for 5 min, and stored at −80°C until analysis. Thawed cell lysates were clarified by centrifugation at 34,000 × *g* for 10 min at 4°C. Ten microliters was used as the input; 100 μL was incubated with 5 μg of the control anti-goat IgG antibody-conjugated protein A/G magnetic beads, and 100 μL was incubated with 5 μg of anti-TIAR antibody-conjugated protein A/G magnetic beads in RIP Immunoprecipitation Buffer (Millipore) at 4°C overnight. After washing with RIP Wash Buffer (Millipore), the beads were recovered with a magnetic separator. The bead samples and the input sample were then incubated at 55°C for 30 min in Proteinase K Buffer (Millipore). The beads were removed by centrifugation, and RNA was extracted using TRI Reagent (Molecular Research Center), dissolved in 20 μL of RNase-free water. The terminal regions of the viral plus-strand and minus-strand RNAs, as well as a control cellular mRNA, GAPDH 3ʹ UTR, were amplified by RT-PCR using the SuperScript III One Step RT-PCR system with Platinum Taq (Invitrogen). The sequences of the primers used are listed in Table S1. One of the specific primers and 1 μL of extracted cell RNA were combined with the RT enzyme mix and incubated at 50°C for 30 min. The RT enzyme was inactivated at 94°C for 10 min, the reverse primer was added, and a 25-cycle PCR was performed. The RT-PCR products were analyzed on 1.2% agarose gels, and the band intensities were quantified using Multi-Gauge V3.1 software. The intensity of the 10% input band for each sample was set at 100%, and the intensities of the immunoprecipitated bands for the same sample were normalized to the input.

### Western blotting

A549 or A549-TIAR cells were grown to ~95% confluency in 6-well plates and then mock-infected or infected with WNV at an MOI of 0.1. At various time points after infection, cells were washed with PBS and lysed by the addition of 150 μL of cell lysis buffer (1% NP-40, 0.1% SDS, 0.5% sodium deoxycholate, and Halt protease and phosphatase inhibitor single-use cocktail [Thermo Scientific] in PBS). Denatured proteins were separated by 10% SDS-PAGE and transferred to a nitrocellulose membrane. The membrane was blocked with 5% non-fat milk in TBS-T buffer (Tris-buffered saline [TBS] buffer [50 mM Tris and 150 mM NaCl, pH 8.0] plus 0.05% of Tween 20) for 1 h at RT and then incubated with the primary antibodies, anti-NS3 (1:1,000, R&D Systems), anti-HA (1:1,000, BioLegend), anti-eIF2α (1:1,000, Cell Signaling), anti-p-eIF2α S51 (1:1,000, Cell Signaling), or anti-β-actin (1:5,000, Abcam), for 1 h at RT in TBS-T buffer containing 5% milk or 5% bovine serum albumin for the anti-p-eIF2α antibody. The membranes were washed and incubated for 1 h at RT in TBS-T buffer containing 5% milk with the secondary antibodies, horseradish peroxidase (HRP)-conjugated anti-rabbit and anti-mouse IgG (Cell Signaling) or HRP-conjugated anti-goat IgG (Santa Cruz Biotechnology). After washing, membranes were processed for enhanced chemiluminescence using a Super-Signal West Pico detection kit (Pierce) according to the manufacturer’s instructions.

### Real-time quantitative RT-PCR

Confluent (~95%) monolayers of A549 or A549-TIAR cells in 6-well plates were mock-infected or infected with WNV (MOI of 0.1 or 5). Cells were washed with PBS, lysed by the addition of TRI Reagent (Molecular Research Center), and then total cell RNA was extracted according to the manufacturer’s protocol. WNV genome RNA was detected using previously designed WNV NS1 region-specific primers and probe (58). β-Actin mRNA was used as the endogenous control and detected with human β-actin primers and probe (Applied Biosystems). These RNAs were assayed in triplicate using a TaqMan one-step RT-PCR master mix reagent kit (Applied Biosystems) and an Applied Biosystems 7500 Fast real-time PCR system according to the manufacturer’s protocol. The cycling parameters were 48°C for 30 min, 95°C for 10 min, 40 cycles at 95°C for 15 s, and 60°C for 1 min. The Ct values obtained for samples harvested at different hpi were normalized to those for β-actin in the same sample and are presented as the relative fold change compared to the 2- or 8-h sample value for the same cell line in relative quantification (RQ) units using the comparative Ct (ΔΔCt) method of the SDS Applied Biosystems software, which also applied statistical analysis to the data. The data were exported and plotted in Microsoft Excel.

To separately quantify intracellular viral plus-strand or minus-strand RNA levels, T7-tagged primer qRT-PCR was performed as previously described (59) using a TaqMan RNA-to-Ct 1-Step kit (Applied Biosystems). Total cell RNA was extracted from monolayers of A549 or A549-TIAR cells infected with WNV at an MOI of 5. The specific primers and probe were designed for the WNV Eg101 NS1 region (Table S1). For viral plus-strand RNA detection, the T7-NS1-Rev primer was used for the RT reaction performed at 48°C for 30 min, and then the T7 primer 5ʹ-GCGTAATACGACTCACTATA-3ʹ, the NS1-For primer, and the NS1 probe 5ʹ 6-FAM-GCACTTGGCCTGAAACGCACACTTTGT-TAMRA-3ʹ were added for the real-time PCR step after RT inactivation at 95°C for 15 min. For viral minus-strand RNA detection, the T7-NS1-For primer was used for the RT reaction, and to add the T7 sequence, the NS1-Rev primer and the NS1 probe were used for the real-time PCR step. Real-time PCR was performed as follows: 40 cycles of 95°C for 15 s and 60°C for 1 min. To calculate the increase in viral plus-strand RNA levels, the amount at 2 h was subtracted from the amount obtained at each subsequent hpi tested. To calculate the increase in viral minus-strand RNA levels, the amount at 12 h was subtracted from the amount obtained at each subsequent hpi tested. The ratio of viral plus-strand RNA to minus-strand RNA was then calculated. The significance of the difference between the ratios obtained for each of the two cell lines was analyzed by the Student’s *t*-test (*P* < 0.05) using Microsoft Excel.

### RNA oligos

The RNA oligos rC_5_ (5ʹ-CCCCC-3ʹ) and rA_5_ (5ʹ-AAAAA-3ʹ) were purchased from Integrated DNA Technologies, Inc. The 2-hydroxyl protected rU_5_ (5ʹ-UUUUU-3ʹ), rU_15_ (5ʹ-UUUUUUUUUUUUUUU-3ʹ), rU_20_ (5ʹ-UUUUUUUUUUUUUUUUUUUU-3ʹ), WNV20 (5ʹ- UUAAUUGUUGUUAAUCCUCA-3ʹ), and 5ʹ fluorescein-labeled rU_20_ (FrU_20_) and rU_6_ (FrU_6_) oligos were purchased from Dharmacon and Sigma Aldrich. The 2-hydroxyl-protected RNA samples were deprotected following the manufacturer’s protocol, desalted using a HiTrap desalting column (GE Healthcare), and lyophilized.

### Isothermal titration calorimetry

ITC experiments were conducted using a VP-ITC microcalorimeter (MicroCal, LLC, Northampton, MA) at 25°C with a reference power of 18 μcal/s and a stirring speed of 351 rpm. Protein samples were dialyzed overnight in D-Tube Dialyzers MWC 6–8 kD (EMD Millipore) against 20 mM PBS, pH 7.0, and 100 mM NaCl and adjusted to the desired concentration. Lyophilized RNA was resuspended in protein dialysis buffer to ensure buffer matching. Samples were degassed prior to loading. Titrations were performed by injecting 8–10 μL into the sample cell every 300 s for a total of 25 injections. The normalized heat signals were calculated using the bundled Origin software, and the data were fitted using a one-site binding model.

### Nuclear magnetic resonance spectroscopy

Data were collected on a Bruker 500 MHz NMR equipped with a 5 mm TBI probe. Protein samples were diluted with NMR sample buffer (10 mM PBS, 50 mM NaCl, 0.1 mM EDTA, and 10% D_2_O) to achieve a final concentration between 80 and 100 µM. Samples were adjusted to pH 6.8. ^1^H spectra were acquired using a pulse program with watergate solvent suppression (zgprw5). The rU_15_T samples were resuspended in 10 mM PBS, 50 mM NaCl, and 0.2 mM EDTA with 10% D_2_O, pH 6.8. RRM123 was added at 1:1, 2:1, or 3:1 ratio, and the pH was adjusted to match the free RNA sample (50 µM). TOCSY data were collected using a pulse program with Watergate solvent suppression (mlevgpphw5) at 20°C with a relaxation time of 2 s and a mixing time of 50 ms.

### Steady-state fluorescence anisotropy

Fluorescence anisotropy experiments were carried out on a Perkin Elmer LS55 fluorescence spectrometer (Waltham, MA) using an excitation and emission wavelength of 495 nm and 521 nm and slit widths of 10 mm and 3 mm, respectively. FrU_20_ and FrU_6_ were prepared in 500 μL of buffer (5 mM PBS at pH 7.0) with high (120 mM KCl), medium (50 mM KCl), or low salt (15 mM KCl) using a 0.5 cm fluorimeter quartz cuvette. Anisotropy of FrU_20_ was measured after each addition of a protein aliquot at 25°C. The data were analyzed using GraphPad Prism 6.

## Data Availability

All data supporting the results of this study are available within the article and supplemental information. Further inquiries can be directed to the corresponding author.

## AFTER EPUB

*[This article was published on 8 September 2026 without a Present Address for Jeenah Jung. The address was added in the current version, posted on 17 September 2026.]*

## SUPPLEMENTAL MATERIAL

Supplemental figures and tables mbio.01947-26-s0001.pdf Tables S1 to S3; Fig. S1 to S6.

ASM does not own the copyrights to Supplemental Material that may be linked to, or accessed through, an article. The authors have granted ASM a non-exclusive, world-wide license to publish the Supplemental Material files. Please contact the corresponding author directly for reuse.

## References

[1] Petersen LR, Marfin AA. 2002. West Nile virus: a primer for the clinician. Ann Intern Med 137:173–179. doi:10.7326/0003-4819-137-3-200208060-00009 12160365

[2] Hall RA, Scherret JH, Mackenzie JS. 2001. Kunjin virus: an Australian variant of West Nile? Ann N Y Acad Sci 951:153–160. doi:10.1111/j.1749-6632.2001.tb02693.x 11797773

[3] Mackenzie JS, Gubler DJ, Petersen LR. 2004. Emerging flaviviruses: the spread and resurgence of Japanese encephalitis, West Nile and dengue viruses. Nat Med 10:S98–S109. doi:10.1038/nm1144 15577938

[4] Blitvich BJ. 2008. Transmission dynamics and changing epidemiology of West Nile virus. Anim Health Res Rev 9:71–86. doi:10.1017/S1466252307001430 18348742

[5] Hayes EB, Komar N, Nasci RS, Montgomery SP, O’Leary DR, Campbell GL. 2005. Epidemiology and transmission dynamics of West Nile virus disease. Emerg Infect Dis 11:1167–1173. doi:10.3201/eid1108.050289a PMC3320478 16102302

[6] Sambri V, Capobianchi M, Charrel R, Fyodorova M, Gaibani P, Gould E, Niedrig M, Papa A, Pierro A, Rossini G, Varani S, Vocale C, Landini MP. 2013. West Nile virus in Europe: emergence, epidemiology, diagnosis, treatment, and prevention. Clin Microbiol Infect 19:699–704. doi:10.1111/1469-0691.12211 23594175

[7] Kramer LD, Li J, Shi PY. 2007. West Nile virus. Lancet Neurol 6:171–181. doi:10.1016/S1474-4422(07)70030-3 17239804

[8] Lindenbach B, Thiel HJ, Rice CM. 2007. Flaviviridae: the viruses and their replication, p 1101–1151. In Fields virology. Lippincott Williams & Wilkins, Philadelphia, PA.

[9] Lindenbach B, Murray C, Thiel H, Rice C. 2013. Flaviviridae, p 712–746. In Fields virology, vol. 1. Lippincott Williams & Wilkins, Philadelphia, PA.

[10] Welsch S, Miller S, Romero-Brey I, Merz A, Bleck CKE, Walther P, Fuller SD, Antony C, Krijnse-Locker J, Bartenschlager R. 2009. Composition and three-dimensional architecture of the dengue virus replication and assembly sites. Cell Host Microbe 5:365–375. doi:10.1016/j.chom.2009.03.007 PMC7103389 19380115

[11] Gillespie LK, Hoenen A, Morgan G, Mackenzie JM. 2010. The endoplasmic reticulum provides the membrane platform for biogenesis of the flavivirus replication complex. J Virol 84:10438–10447. doi:10.1128/JVI.00986-10 PMC2950591 20686019

[12] Miorin L, Romero-Brey I, Maiuri P, Hoppe S, Krijnse-Locker J, Bartenschlager R, Marcello A. 2013. Three-dimensional architecture of tick-borne encephalitis virus replication sites and trafficking of the replicated RNA. J Virol 87:6469–6481. doi:10.1128/JVI.03456-12 PMC3648123 23552408

[13] Chambers TJ, Hahn CS, Galler R, Rice CM. 1990. Flavivirus genome organization, expression, and replication. Annu Rev Microbiol 44:649–688. doi:10.1146/annurev.mi.44.100190.003245 2174669

[14] Alvarez DE, Lodeiro MF, Ludueña SJ, Pietrasanta LI, Gamarnik AV. 2005. Long-range RNA-RNA interactions circularize the dengue virus genome. J Virol 79:6631–6643. doi:10.1128/JVI.79.11.6631-6643.2005 PMC1112138 15890901

[15] Huber RG, Lim XN, Ng WC, Sim AYL, Poh HX, Shen Y, Lim SY, Sundstrom KB, Sun X, Aw JG, Too HK, Boey PH, Wilm A, Chawla T, Choy MM, Jiang L, de Sessions PF, Loh XJ, Alonso S, Hibberd M, Nagarajan N, Ooi EE, Bond PJ, Sessions OM, Wan Y. 2019. Structure mapping of dengue and Zika viruses reveals functional long-range interactions. Nat Commun 10:1408. doi:10.1038/s41467-019-09391-8 PMC6441010 30926818

[16] Villordo SM, Gamarnik AV. 2009. Genome cyclization as strategy for flavivirus RNA replication. Virus Res 139:230–239. doi:10.1016/j.virusres.2008.07.016 PMC5440119 18703097

[17] Ortín J, Parra F. 2006. Structure and function of RNA replication. Annu Rev Microbiol 60:305–326. doi:10.1146/annurev.micro.60.080805.142248 16719717

[18] Li W, Li Y, Kedersha N, Anderson P, Emara M, Swiderek KM, Moreno GT, Brinton MA. 2002. Cell proteins TIA-1 and TIAR interact with the 3’ stem-loop of the West Nile virus complementary minus-strand RNA and facilitate virus replication. J Virol 76:11989–12000. doi:10.1128/jvi.76.23.11989-12000.2002 PMC136884 12414941

[19] Förch P, Puig O, Kedersha N, Martínez C, Granneman S, Séraphin B, Anderson P, Valcárcel J. 2000. The apoptosis-promoting factor TIA-1 is a regulator of alternative pre-mRNA splicing. Mol Cell 6:1089–1098. doi:10.1016/s1097-2765(00)00107-6 11106748

[20] Kedersha N, Cho MR, Li W, Yacono PW, Chen S, Gilks N, Golan DE, Anderson P. 2000. Dynamic shuttling of TIA-1 accompanies the recruitment of mRNA to mammalian stress granules. J Cell Biol 151:1257–1268. doi:10.1083/jcb.151.6.1257 PMC2190599 11121440

[21] Kedersha NL, Gupta M, Li W, Miller I, Anderson P. 1999. RNA-binding proteins TIA-1 and TIAR link the phosphorylation of eIF-2 alpha to the assembly of mammalian stress granules. J Cell Biol 147:1431–1442. doi:10.1083/jcb.147.7.1431 PMC2174242 10613902

[22] Taupin JL, Tian Q, Kedersha N, Robertson M, Anderson P. 1995. The RNA-binding protein TIAR is translocated from the nucleus to the cytoplasm during fas-mediated apoptotic cell death. Proc Natl Acad Sci USA 92:1629–1633. doi:10.1073/pnas.92.5.1629 PMC42573 7533298

[23] Söderberg O, Gullberg M, Jarvius M, Ridderstråle K, Leuchowius K-J, Jarvius J, Wester K, Hydbring P, Bahram F, Larsson L-G, Landegren U. 2006. Direct observation of individual endogenous protein complexes in situ by proximity ligation. Nat Methods 3:995–1000. doi:10.1038/nmeth947 17072308

[24] Yi Z, Yuan Z, Rice CM, MacDonald MR. 2012. Flavivirus replication complex assembly revealed by DNAJC14 functional mapping. J Virol 86:11815–11832. doi:10.1128/JVI.01022-12 PMC3486285 22915803

[25] Kim HS, Wilce MCJ, Yoga YMK, Pendini NR, Gunzburg MJ, Cowieson NP, Wilson GM, Williams BR, Gorospe M, Wilce JA. 2011. Different modes of interaction by TIAR and HuR with target RNA and DNA. Nucleic Acids Res 39:1117–1130. doi:10.1093/nar/gkq837 PMC3035456 21233170

[26] López de Silanes I, Galbán S, Martindale JL, Yang X, Mazan-Mamczarz K, Indig FE, Falco G, Zhan M, Gorospe M. 2005. Identification and functional outcome of mRNAs associated with RNA-binding protein TIA-1. Mol Cell Biol 25:9520–9531. doi:10.1128/MCB.25.21.9520-9531.2005 PMC1265820 16227602

[27] Mazan-Mamczarz K, Lal A, Martindale JL, Kawai T, Gorospe M. 2006. Translational repression by RNA-binding protein TIAR. Mol Cell Biol 26:2716–2727. doi:10.1128/MCB.26.7.2716-2727.2006 PMC1430315 16537914

[28] Cleaves GR, Ryan TE, Schlesinger RW. 1981. Identification and characterization of type 2 dengue virus replicative intermediate and replicative form RNAs. Virology 111:73–83. doi:10.1016/0042-6822(81)90654-1 6263011

[29] Kim HS, Headey SJ, Yoga YMK, Scanlon MJ, Gorospe M, Wilce MCJ, Wilce JA. 2013. Distinct binding properties of TIAR RRMs and linker region. RNA Biol 10:579–589. doi:10.4161/rna.24341 PMC3710364 23603827

[30] Dember LM, Kim ND, Liu KQ, Anderson P. 1996. Individual RNA recognition motifs of TIA-1 and TIAR have different RNA binding specificities. J Biol Chem 271:2783–2788. doi:10.1074/jbc.271.5.2783 8576255

[31] Bauer WJ, Heath J, Jenkins JL, Kielkopf CL. 2012. Three RNA recognition motifs participate in RNA recognition and structural organization by the pro-apoptotic factor TIA-1. J Mol Biol 415:727–740. doi:10.1016/j.jmb.2011.11.040 PMC3282181 22154808

[32] Cruz-Gallardo I, Aroca Á, Gunzburg MJ, Sivakumaran A, Yoon J-H, Angulo J, Persson C, Gorospe M, Karlsson BG, Wilce JA, Díaz-Moreno I. 2014. The binding of TIA-1 to RNA C-rich sequences is driven by its C-terminal RRM domain. RNA Biol 11:766–776. doi:10.4161/rna.28801 PMC4156507 24824036

[33] Zuker M. 2003. Mfold web server for nucleic acid folding and hybridization prediction. Nucleic Acids Res 31:3406–3415. doi:10.1093/nar/gkg595 PMC169194 12824337

[34] Kielkopf CL, Rodionova NA, Green MR, Burley SK. 2001. A novel peptide recognition mode revealed by the X-ray structure of a core U2AF35/U2AF65 heterodimer. Cell 106:595–605. doi:10.1016/s0092-8674(01)00480-9 11551507

[35] Förch P, Puig O, Martínez C, Séraphin B, Valcárcel J. 2002. The splicing regulator TIA-1 interacts with U1-C to promote U1 snRNP recruitment to 5’ splice sites. EMBO J 21:6882–6892. doi:10.1093/emboj/cdf668 PMC139089 12486009

[36] Emara MM, Liu H, Davis WG, Brinton MA. 2008. Mutation of mapped TIA-1/TIAR binding sites in the 3’ terminal stem-loop of West Nile virus minus-strand RNA in an infectious clone negatively affects genomic RNA amplification. J Virol 82:10657–10670. doi:10.1128/JVI.00991-08 PMC2573169 18768985

[37] Filomatori CV, Lodeiro MF, Alvarez DE, Samsa MM, Pietrasanta L, Gamarnik AV. 2006. A 5’ RNA element promotes dengue virus RNA synthesis on a circular genome. Genes Dev 20:2238–2249. doi:10.1101/gad.1444206 PMC1553207 16882970

[38] Dong H, Zhang B, Shi PY. 2008. Terminal structures of West Nile virus genomic RNA and their interactions with viral NS5 protein. Virology 381:123–135. doi:10.1016/j.virol.2008.07.040 18799181

[39] Pettersen EF, Goddard TD, Huang CC, Couch GS, Greenblatt DM, Meng EC, Ferrin TE. 2004. UCSF Chimera--a visualization system for exploratory research and analysis. J Comput Chem 25:1605–1612. doi:10.1002/jcc.20084 15264254

[40] Waris S, García-Mauriño SM, Sivakumaran A, Beckham SA, Loughlin FE, Gorospe M, Díaz-Moreno I, Wilce MCJ, Wilce JA. 2017. TIA-1 RRM23 binding and recognition of target oligonucleotides. Nucleic Acids Res 45:4944–4957. doi:10.1093/nar/gkx102 PMC5416816 28184449

[41] Brown A. 2009. Analysis of cooperativity by isothermal titration calorimetry. Int J Mol Sci 10:3457–3477. doi:10.3390/ijms10083457 PMC2812830 20111687

[42] Cieniková Z, Jayne S, Damberger FF, Allain F-T, Maris C. 2015. Evidence for cooperative tandem binding of hnRNP C RRMs in mRNA processing. RNA 21:1931–1942. doi:10.1261/rna.052373.115 PMC4604433 26370582

[43] Loughlin FE, West DL, Gunzburg MJ, Waris S, Crawford SA, Wilce MCJ, Wilce JA. 2021. Tandem RNA binding sites induce self-association of the stress granule marker protein TIA-1. Nucleic Acids Res 49:2403–2417. doi:10.1093/nar/gkab080 PMC7969032 33621982

[44] Stitzinger SH, Sohrabi-Jahromi S, Söding J. 2023. Cooperativity boosts affinity and specificity of proteins with multiple RNA-binding domains. NAR Genom Bioinform 5:lqad057. doi:10.1093/nargab/lqad057 PMC10251633 37305168

[45] Kao CC, Del Vecchio AM, Zhong W. 1999. De novo initiation of RNA synthesis by a recombinant flaviviridae RNA-dependent RNA polymerase. Virology 253:1–7. doi:10.1006/viro.1998.9517 9887312

[46] Selisko B, Potisopon S, Agred R, Priet S, Varlet I, Thillier Y, Sallamand C, Debart F, Vasseur JJ, Canard B. 2012. Molecular basis for nucleotide conservation at the ends of the dengue virus genome. PLoS Pathog 8:e1002912. doi:10.1371/journal.ppat.1002912 PMC3441707 23028313

[47] Abram QH, Landry BN, Wang AB, Kothe RF, Hauch HCH, Sagan SM. 2024. The myriad roles of RNA structure in the flavivirus life cycle. RNA Biol 21:14–30. doi:10.1080/15476286.2024.2357857 PMC11135854 38797925

[48] Lee E, Bujalowski PJ, Teramoto T, Gottipati K, Scott SD, Padmanabhan R, Choi KH. 2021. Structures of flavivirus RNA promoters suggest two binding modes with NS5 polymerase. Nat Commun 12:2530. doi:10.1038/s41467-021-22846-1 PMC8100141 33953197

[49] Tipo J, Gottipati K, Choi KH. 2024. High-resolution RNA tertiary structures in zika virus stem-loop A for the development of inhibitory small molecules. RNA 30:609–623. doi:10.1261/rna.079796.123 PMC11098461 38383158

[50] Knyazhanskaya E, Bujalowski PJ, Le MT, Gottipati K, Choi KH. 2026. Interactions of dengue virus NS5 and NS3 with the 3’ end of Its negative-strand RNA. Viruses 18:226. doi:10.3390/v18020226 PMC12945261 41754569

[51] Nagy PD, Bujarski JJ. 1997. Engineering of homologous recombination hotspots with AU-rich sequences in brome mosaic virus. J Virol 71:3799–3810. doi:10.1128/JVI.71.5.3799-3810.1997 PMC191530 9094655

[52] Noueiry AO, Chen J, Ahlquist P. 2000. A mutant allele of essential, general translation initiation factor DED1 selectively inhibits translation of a viral mRNA. Proc Natl Acad Sci USA 97:12985–12990. doi:10.1073/pnas.240460897 PMC27165 11069307

[53] Jung J, Lifland AW, Alonas EJ, Zurla C, Santangelo PJ. 2013. Characterization of mRNA-cytoskeleton interactions in situ using FMTRIP and proximity ligation. PLoS One 8:e74598. doi:10.1371/journal.pone.0074598 PMC3770708 24040294

[54] Yakhnin AV, Yakhnin H, Babitzke P. 2012. Gel mobility shift assays to detect protein-RNA interactions. Methods Mol Biol 905:201–211. doi:10.1007/978-1-61779-949-5_12 PMC4687016 22736005

[55] Gagnon KT, Maxwell ES. 2011. Electrophoretic mobility shift assay for characterizing RNA-protein interaction. Methods Mol Biol 703:275–291. doi:10.1007/978-1-59745-248-9_19 21125497

[56] Ryder SP, Recht MI, Williamson JR. 2008. Quantitative analysis of protein-RNA interactions by gel mobility shift. Methods Mol Biol 488:99–115. doi:10.1007/978-1-60327-475-3_7 PMC2928675 18982286

[57] Daugherty MD, D’Orso I, Frankel AD. 2008. A solution to limited genomic capacity: using adaptable binding surfaces to assemble the functional HIV Rev oligomer on RNA. Mol Cell 31:824–834. doi:10.1016/j.molcel.2008.07.016 PMC2651398 18922466

[58] Scherbik SV, Paranjape JM, Stockman BM, Silverman RH, Brinton MA. 2006. RNase l plays a role in the antiviral response to West Nile virus. J Virol 80:2987–2999. doi:10.1128/JVI.80.6.2987-2999.2006 PMC1395436 16501108

[59] Davis WG, Blackwell JL, Shi PY, Brinton MA. 2007. Interaction between the cellular protein eEF1A and the 3’-terminal stem-loop of West Nile virus genomic RNA facilitates viral minus-strand RNA synthesis. J Virol 81:10172–10187. doi:10.1128/JVI.00531-07 PMC2045417 17626087

