# Supplementary material for: The cellular protein TIAR mediates rapid initiation of West Nile virus genome RNA synthesis
Source: mBio. 2026 Sep 8;17(10):e01947-26. doi: 10.1128/mbio.01947-26 (PMC13712925; doi:10.1128/mbio.01947-26)
Supplement: Supplementary file 1 [file mbio.01947-26-s0001.pdf]

Supplementary Table 1. RT-PCR Primer Sequences.

Target	Primer sequence
WNV 3' (-) SL RNA	NCR75F: 5'-AGTAGTTCGCCTGTGTGAGC-3'<br>NCR75R: 5'-[T7] [a] CAGCTCGCACCGTGTTAATTGTTG-3'
WNV 5' (+) SL RNA	NCR75R: 5'-[T7]CAGCTCGCACCGTGTTAATTGTTG-3'<br>NCR75F: 5'-AGTAGTTCGCCTGTGTGAGC-3'
WNV 3' (+) SL RNA	3'F RNA: 5'-[T7]CCTGGGATAGACTAGGAGATCTTCTGC-3'<br>3'R RNA: 5'-AGTATCCTGTGTTCTCGCACCACC-3'
WNV 5' (-) SL RNA	3'R RNA: 5'-AGTATCCTGTGTTCTCGCACCACC-3'<br>3'F RNA: 5'-[T7]CCTGGGATAGACTAGGAGATCTTCTGC-3'
GAPDH 3' UTR	GAPDH 3'UTR R: 5'-GGTTGAGCACAGGGTACTTTATT G-3'<br>GAPDH 3'UTR F: 5'-GACCCCTGGACCACCAGCC-3'
WNV NS1	NS1-For: 5′-GGCGGTCCTGGGTGAAGTCAA-3′<br>T7-NS1-For: 5′-[T7] GGCGGTCCTGGGTGAAGTC-3′<br>T7-NS1-Rev: 5′-[T7]CTCCGATTGTGGTTGCTTC-3′<br>NS1-Rev: 5′-CTCCGATTGTGGTTGCTTCGT-3′

[a] [T7]: TAATACGACTCACTATAGG

Supplementary Table 2. Thermodynamic and stoichiometric parameters from fitted ITC binding curves.

Protein	RNA	n	ΔH (kcal/mol)	TΔS(kcal/mol)	ΔG (kcal/mol)
TIAR	rU_{20}	0.54 ± 0.01	-83.6 ± 1.7 [a]	-74.5 [b]	-9.1
	WNV20	0.37 ± 0.01	-39.1 ± 1.0 [a]	-29.7 [b]	-9.4
RRM123	rU_5	0.85 ± 0.28	-25.8 ± 9.7 [c]	-19.0	-6.8
	rU_{15}	0.55 ± 0.01	-57.0 ± 0.5	-46.5	-10.3
	rU_{20}	0.40 ± 0.01	-84.4 ± 8.0 [a, c]	-75.1 [b]	-9.2
	WNV20	0.40 ± 0.01	-41.4 ± 1.7 [a, c]	-31.9 [b]	-9.5
	L3	0.56 ± 0.02	-32.4 ± 1.4	-24.0	-8.4
	L1+L2	0.47 ± 0.03	-9.4 ± 0.9	-0.9	-8.5
	3′(-) SL	0.31 ± 0.01	-15.5 ± 0.6	-5.8	-9.7
RRM2	rU_5	0.89 ± 0.01	-24.0 ± 0.3 [c]	-17.7	-6.9
	rU_{15}	0.52 ± 0.01	-45.0 ± 1.6	-36.1	-8.8
	rU_{20}	0.34 ± 0.01	-53.6 ± 0.7 [c]	-44.7	-9.0
	WNV20	0.30 ± 0.01	-12.7 ± 0.5 [c]	-4.0	-8.7

[a] The enthalpies for the TIAR and RRM123 titrations with rU_{20} or WNV20, reveal a trend where the enthalpies for the WNV20 titrations were approximately half of the enthalpies for the rU_{20} titrations. We attribute these reductions in enthalpy to fewer RNA-protein contacts since ten bases in the WNV20 sequence are not U residues.

[b] In the titrations with TIAR or RRM123 with WNV20 or rU_{20}, the calculated entropies are much higher for rU_{20}. This may be caused by fewer RNA-protein contacts in WNV20 complexes as compared to the rU_{20} complexes. The resulting increased flexibility may minimize protein-protein electrostatic repulsion and could explain the increased affinities of TIAR and RRM123 with the WNV20 sequence compared to the rU_{20} sequence (Table 2).

[c] The enthalpies for rU20 or WNV with RRM2 are both ~30 kcal/mol less than the enthalpies for RRM123 titrated with the same sequences; while there is no significant difference in ΔH values for the rU5 sequence with RRM2 and RRM123 where there is a single copy of protein. Given that the differences in ΔH are sequence independent for the 20 nt RNAs and only occur when there are multiple proteins bound, we conclude that the additional energy must result from RRM1/RRM3 interactions between proteins.

Supplementary Table 3. Competition between FrU_6 and rU_5.

FrU_6 [μM]	RRM2 [μM]	rU5 [μM]	Anisotropy
0.250	2.50	0	0.200
0.250	3.00	0	0.205
0.250	3.50	0	0.207
0.250	3.50	0.250	0.206
0.250	3.50	0.500	0.204

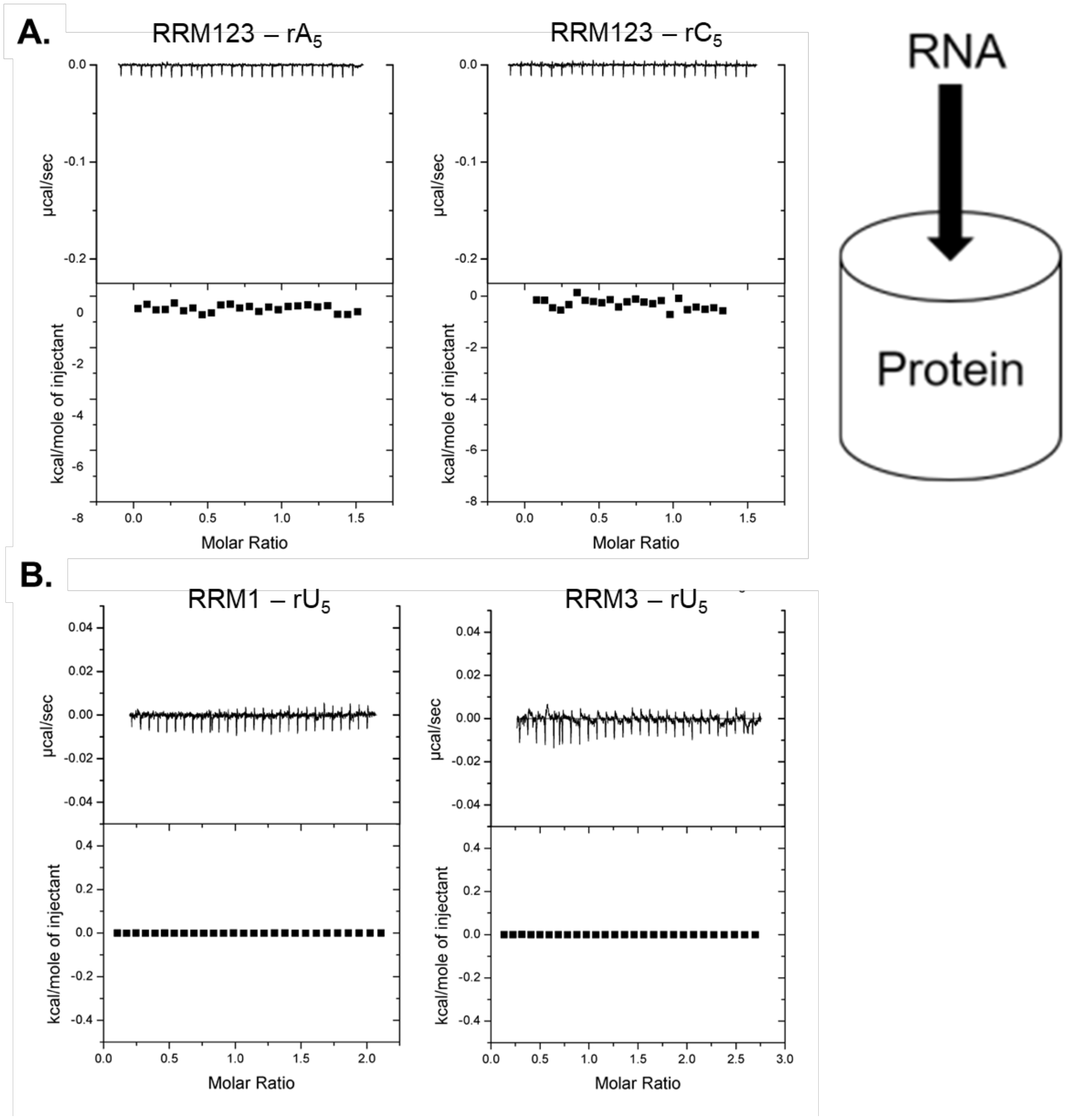

Supplementary Figure 1. Nonbinding and low affinity isotherms. (A) RRM123 (5 µM) with rA_5 or rC_5 RNA (50 µM) was titrated into protein. All titrations were performed in 20 mM sodium phosphate buffer with 100 mM NaCl. (B) RRM1 or RRM3 (5 µM) with rU_5(50 µM), a previously identified high affinity substrate for TIAR RRM123.

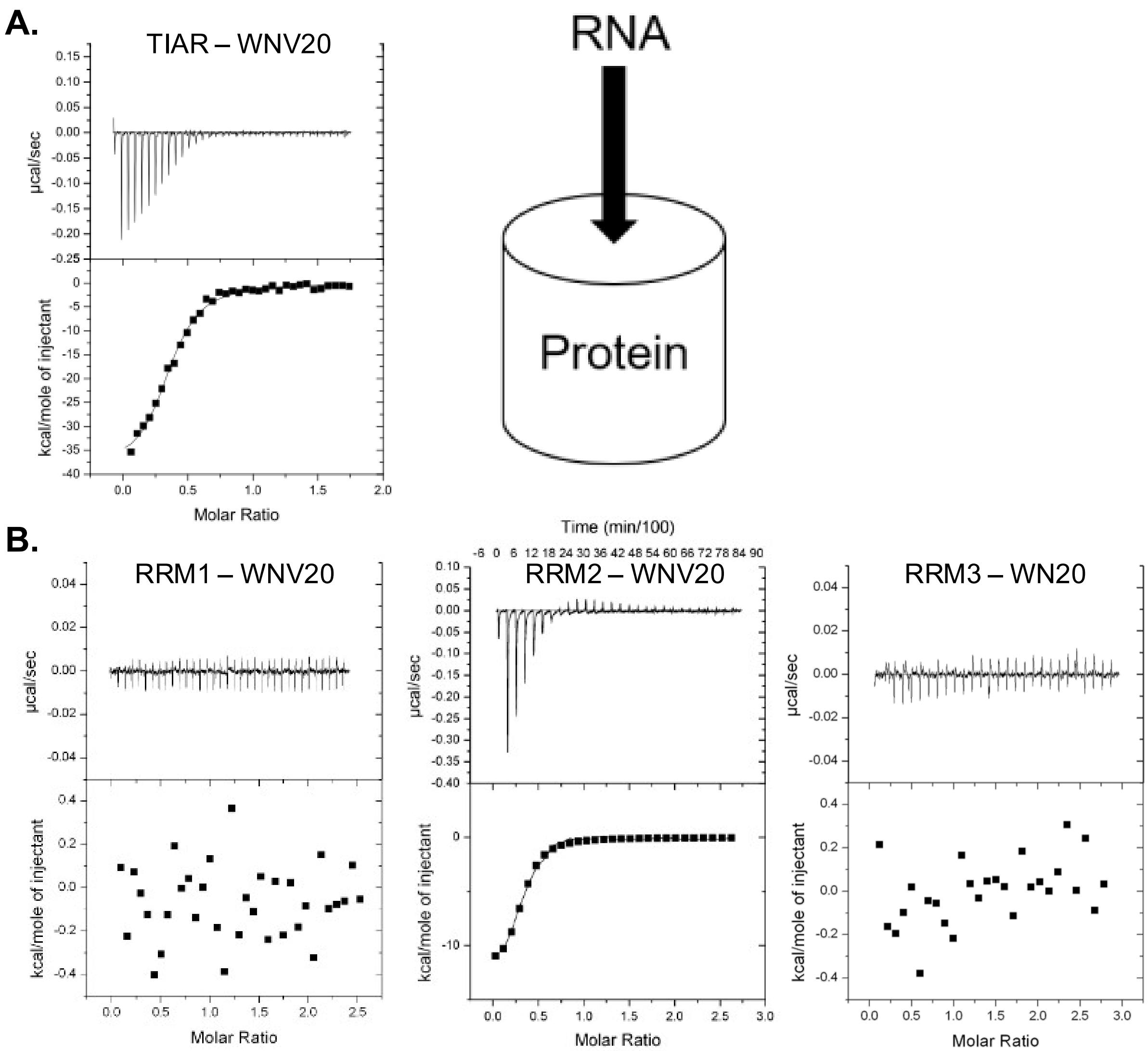

Supplementary Figure 2. ITC with the WNV20 RNA oligo. WNV20 RNA (~30-50 µM) was titrated into (~3-5 µM) of (A) full length TIAR or individual (B) RRMs. All titrations were performed in 20 mM sodium phosphate buffer with 100 mM NaCl.

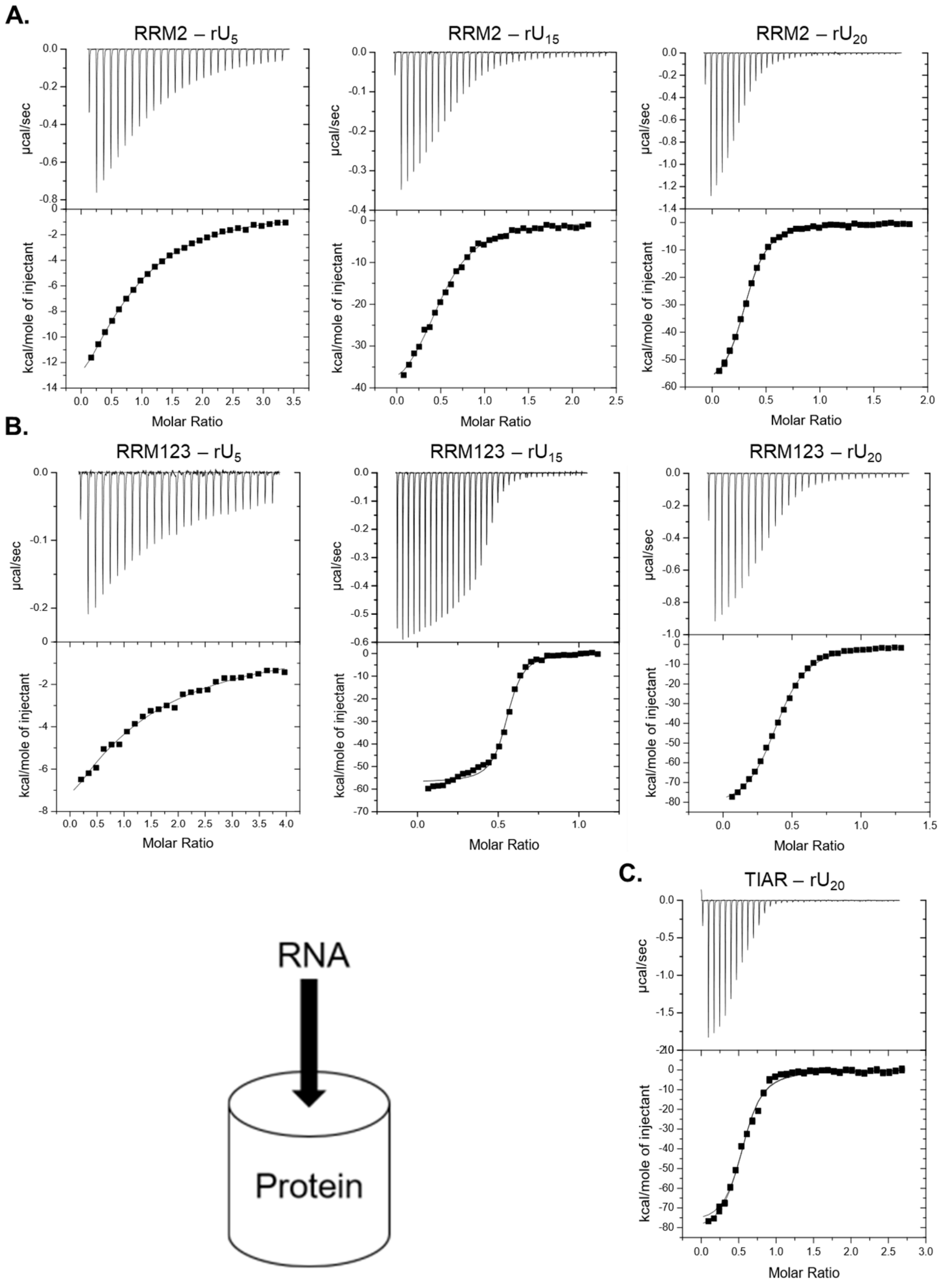

Supplementary Figure 3. Isotherms and fitted binding curves for rU_n sequences with RRM2 and RRM123. 3-7 µM of (A) RRM2 (B) RRM123 or (C) TIAR was titrated with 30-100 µM of a rU_n oligo (where n is the length) in 20 mM sodium phosphate buffer with 100 mM NaCl. The isotherms obtained with the rU5, rU15 and rU20 RNAs were fit with one-site independent binding models using the Origin MicroCal software.

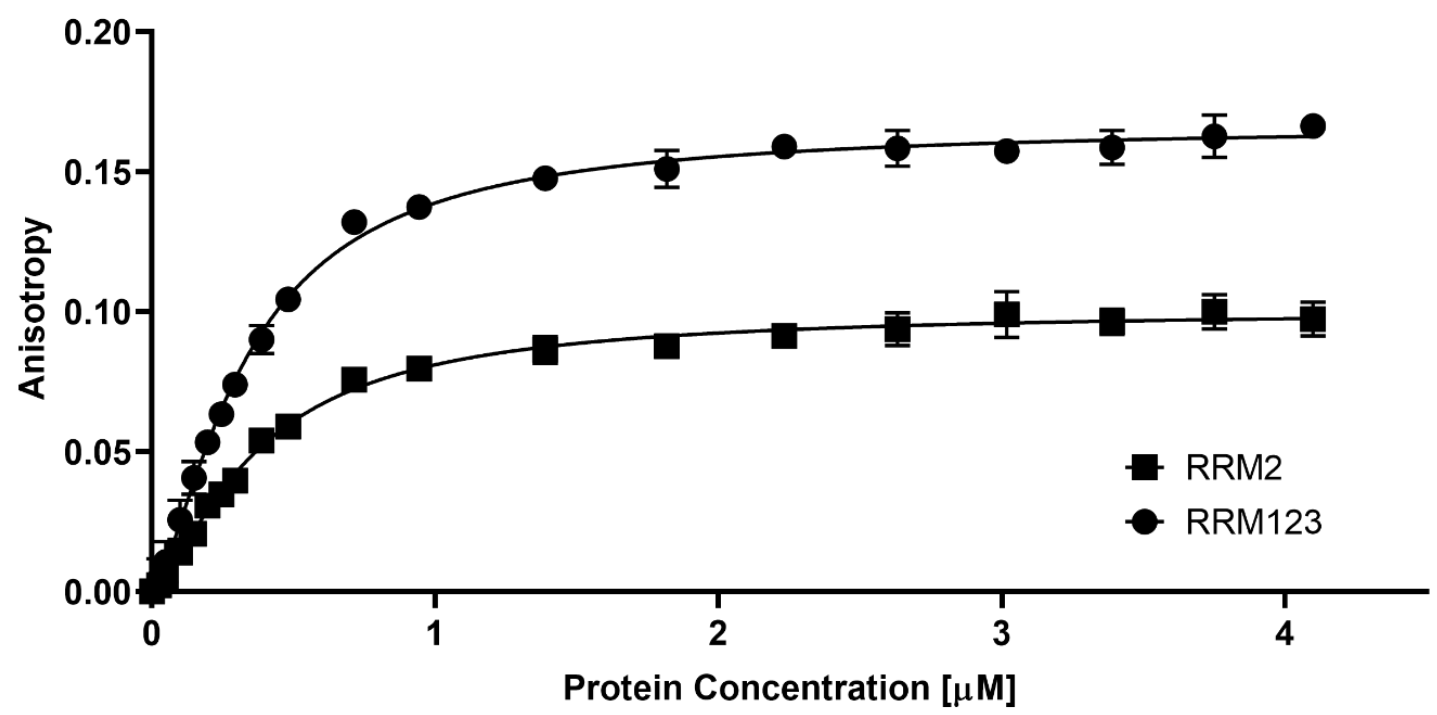

Supplementary Figure 4. Fluorescence anisotropy of FrU20. FrU20 (250 nM) was titrated with increasing concentrations of RRM2 (squares) or RRM123 (circles) in 5 mM sodium phosphate buffer with 15 mM KCl. Binding was monitored using steady-state fluorescence anisotropy at RT. Measurements were performed in triplicate and error bars represent SD.

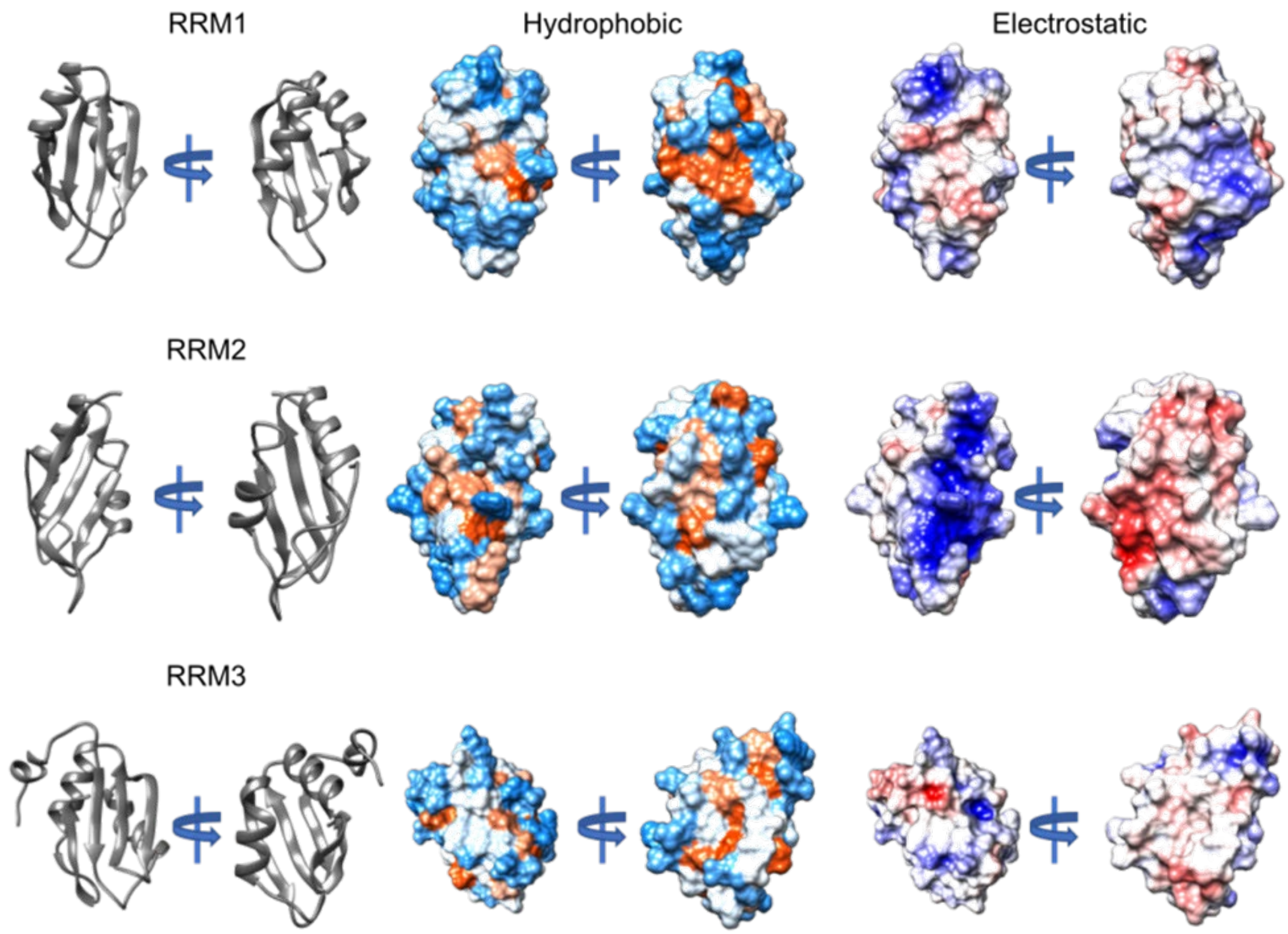

Supplementary Figure 5. Hydrophobic and electrostatic surface maps of the TIAR RRMs. Ribbon representation, hydrophobic and electrostatic maps of each RRM subunit were generated using UCSF Chimera. For the hydrophobic maps, orange indicates hydrophobic and blue indicates polar patches. Electrostatic maps are from +5eV (blue) to -5eV (red). Left sides: bottom view, into the β-sheet; right sides: top view into the α helices. Only the RNA binding surface of RRM2 is highly charged. [PDB IDs: RRM1 2CQI; RRM2 2DH7; RRM3 1X4G]

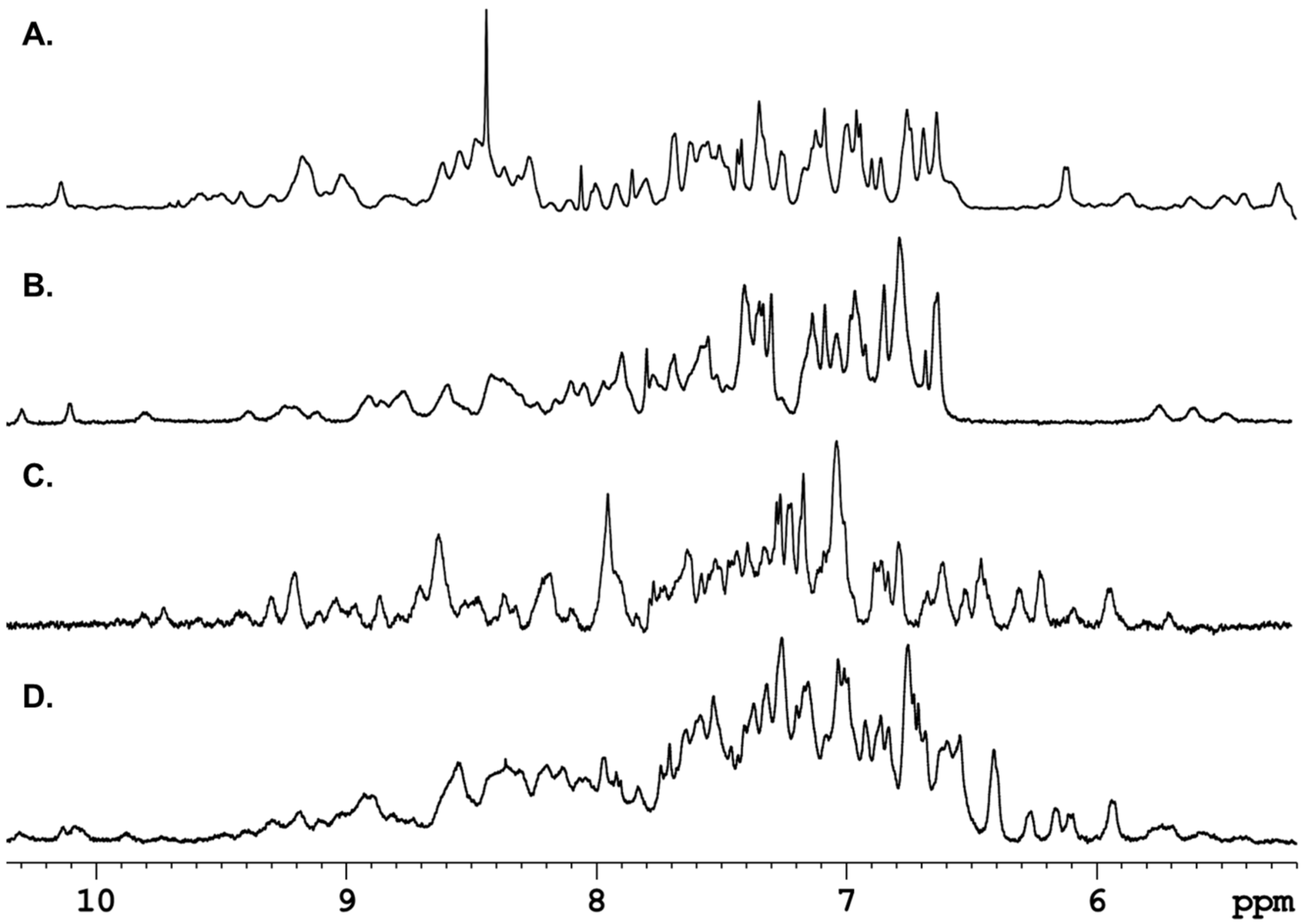

Supplementary Figure 6. Downfield ^{1}H NMR region of RRM1, RRM2, RRM3 and RRM123. Spectra for A) RRM1, B) RRM2, C) RRM3, or D) RRM123 (80-100μM) were acquired in 10 mM PBS, 50 mM NaCl, 1 mM EDTA pH 6.8 and 10% D_2O using a pulse program with watergate solvent suppression (zgprw5).
